# Phytochemical Profiling and *α*-Glucosidase and *α*-Amylase Inhibitory Activities of Underutilised *Nanhaia speciosa* Aerial Biomass

**DOI:** 10.3390/foods15173167

**Published:** 2026-09-07

**Authors:** Wei Dai, Jing Yang, Yiting Chen, Liangqian Zhang, Yuxi Wu, Gantao Cheng, Qi Wang, Xin He

**Affiliations:** 1 Teaching and Experimental Center, Guangdong Pharmaceutical University, Guangzhou 510006, China; dai_gdpu_2018@gdpu.edu.cn; 2 Comprehensive Experimental Teaching Center of Traditional Chinese Medicine, Yunfu Campus, Guangdong Pharmaceutical University, Yunfu 527500, China; 3 School of Traditional Chinese Materia Medica, Guangdong Pharmaceutical University, Guangzhou 510006, China; 4 Key Laboratory of Xinjiang Phytomedicine Resource and Utilization, Ministry of Education, School of Pharmacy, Shihezi University, Shihezi 832002, China; 5 Guangdong Xiaoyang Ecological Agriculture Co., Ltd., Yunfu 527500, China

**Keywords:** *Nanhaia speciosa*, by-product valorisation, bioactive phytochemicals, *α*-glucosidase, *α*-amylase, molecular dynamics simulation

## Abstract

The aerial parts of *Nanhaia speciosa* are often discarded despite their potential as a source of bioactive compounds. This study aimed to characterise this underutilised biomass and evaluate its functional properties relevant to carbohydrate digestion. Ultra-high-performance liquid chromatography–quadrupole-Orbitrap high-resolution mass spectrometry (UHPLC-Q-Orbitrap HRMS), Global Natural Products Social (GNPS) molecular networking, and MS/MS fragmentation analysis tentatively annotated 96 compounds, mainly flavonoids and nitrogen-containing constituents. The extract inhibited *α*-glucosidase and *α*-amylase in a concentration-dependent manner, with IC_50_ values of 9.98 ± 0.02 and 174.83 ± 1.31 μg/mL, respectively, compared with 29.73 ± 0.04 and 39.46 ± 0.19 μg/mL for acarbose. The extract also showed measurable chemical antioxidant capacity. Network pharmacology suggested potential involvement of cAMP-, calcium-, and receptor-associated signalling. Molecular docking prioritised butein as a candidate constituent with favourable predicted interactions with both enzymes, and molecular dynamics simulations supported stable predicted binding. The estimated MM/GBSA binding free energies of butein were −36.68 and −32.03 kcal/mol for *α*-glucosidase and *α*-amylase, respectively. These findings indicate that the aerial biomass of *N. speciosa* is a promising source of phytochemicals with potential carbohydrate-hydrolase inhibitory properties, although the activity of individual constituents requires further experimental validation.

## 1. Introduction

Valorisation of underutilised plant biomass is an important strategy for improving resource efficiency and recovering bioactive constituents with potential value for food and nutraceutical applications. This is particularly relevant for medicinal and edible plants in which selected organs are conventionally utilised while substantial amounts of other tissues remain unused or are discarded. One health-related application of such plant-derived bioactives is the modulation of postprandial carbohydrate digestion. Postprandial hyperglycaemia is an important metabolic feature of type 2 diabetes mellitus and is closely associated with the digestion and absorption of dietary carbohydrates. According to the International Diabetes Federation, diabetes affected approximately 589 million adults aged 20–79 years in 2024 and is projected to affect 853 million by 2050 [1]. During carbohydrate digestion, pancreatic *α*-amylase hydrolyses starch into smaller oligosaccharides, which are subsequently converted into absorbable glucose by intestinal brush-border *α*-glucosidases, particularly maltase-glucoamylase and sucrase-isomaltase [2]. Modulation of these carbohydrate-hydrolysing enzymes can therefore delay glucose release and reduce postprandial glycaemic excursions. Although acarbose and related inhibitors are clinically effective, excessive inhibition of carbohydrate digestion may be accompanied by gastrointestinal adverse effects. Consequently, increasing attention has been directed towards plant-derived constituents with differentiated inhibitory effects on *α*-glucosidase and *α*-amylase [3,4].

Medicinal and edible plants contain structurally diverse constituents, including flavonoids, phenolic acids, tannins, alkaloids, and terpenoids, that may contribute to carbohydrate-hydrolase inhibition [3,4]. Flavonoids are particularly relevant because their hydroxylation pattern, C-ring unsaturation, glycosylation, and molecular size can influence their interactions with digestive enzymes [3]. Plant-derived phenolic constituents may also provide complementary antioxidant properties, which are potentially relevant because oxidative stress contributes to β-cell dysfunction, insulin resistance, and diabetic complications [5]. Accordingly, the recovery and characterisation of such compounds from underused plant materials may provide both a resource-valorisation opportunity and a basis for identifying candidate bioactive ingredients.

*Nanhaia speciosa* (Champ. ex Benth.) J. Compton & Schrire is a perennial climbing plant belonging to the Fabaceae. The species has frequently been reported under the former names *Millettia speciosa* Champ. and *Callerya speciosa* (Champ. ex Benth.) Schot, whereas *N. speciosa* is the currently accepted taxonomic name following the redefinition of the Callerya group and tribe Wisterieae [6]. Its tuberous roots, commonly known as Niu dali in southern China, are traditionally consumed in soups, herbal preparations, and medicinal wines. Previous investigations have reported polysaccharides, flavonoids, isoflavonoids, alkaloids, sterols, and other specialised metabolites in the roots, and a chemically characterised root extract has been shown to ameliorate disturbances in glucose and lipid metabolism [7]. In contrast to the extensively utilised roots, the stems, branches, and leaves represent a considerable proportion of the plant biomass but remain poorly utilised. Comparative analysis of different tissues has demonstrated that the leaves and branches contain abundant nutrients and functional metabolites, suggesting value beyond disposal as residual plant material [8]. More recently, the aerial parts were explicitly reported to be frequently discarded, leading to resource waste; 41 constituents, mainly flavonoids and alkaloids, were detected in the stems, branches, and leaves, and the representative isoflavonoid formononetin was further investigated for its potential metabolic effects [9]. Our previous work also established a standard-confirmed UPLC-Q-Orbitrap HRMS method for hypaphorine in the roots of *N. speciosa* and developed an ultrasound-assisted deep eutectic solvent extraction-macroporous resin process for its efficient recovery [10]. In addition, root extracts and fractions have exhibited *α*-glucosidase and *α*-amylase inhibitory activities before and after simulated gastrointestinal digestion [11]. Together, these findings indicate that the discarded aerial biomass may retain chemically and functionally valuable constituents and merits systematic evaluation as a plant-derived by-product resource.

Despite these advances, the phytochemical diversity of the aerial parts of *N. speciosa* has not been comprehensively characterised in relation to their direct inhibitory activities against *α*-glucosidase and *α*-amylase. Previous investigations have largely focused on nutritional composition, selected compound classes, or individual representative constituents, and the chemical basis potentially underlying carbohydrate-hydrolase inhibition remains insufficiently defined. High-resolution liquid chromatography–tandem mass spectrometry provides an effective approach for the comprehensive characterisation of complex botanical extracts through accurate precursor masses, isotope patterns, and product-ion information. Global Natural Products Social (GNPS) molecular networking further facilitates spectral-library matching, dereplication, and the organisation of structurally related metabolites into molecular families [12,13]. Integration of LC–HRMS/MS profiling with direct enzyme-inhibition assays therefore provides an opportunity to link chemical composition with experimentally observed bioactivity. Complementary computational approaches, including molecular docking [14,15,16,17] and molecular dynamics simulations [18,19], can subsequently be used to prioritise experimentally detected constituents and assess the plausibility and stability of predicted enzyme–ligand interactions. Network pharmacology may additionally generate hypotheses regarding broader compound–target and pathway associations [20,21]. However, such computational evidence does not establish direct target engagement or therapeutic efficacy and should be interpreted as supportive and hypothesis-generating rather than confirmatory [22].

Accordingly, we hypothesised that the underutilised aerial biomass of *N. speciosa* contains structurally diverse phytochemicals associated with carbohydrate-hydrolase inhibitory activity. This study combined UHPLC-Q-Orbitrap HRMS, GNPS molecular networking, and representative MS/MS fragmentation analysis to characterise the phytochemical profile of the aerial parts; evaluated the extract for *α*-glucosidase and *α*-amylase inhibition together with complementary chemical antioxidant capacity; and applied network pharmacology, molecular docking, and molecular dynamics simulations to prioritise potentially relevant constituents and explore plausible molecular interactions. By integrating chemical characterisation, experimental bioactivity, and computational prioritisation, the study aimed to provide a scientific basis for the value-added utilisation of a frequently discarded plant biomass and to identify candidate bioactive phytochemicals for subsequent validation and potential ingredient-oriented development.

## 2. Materials and Methods

### 2.1. Plant Material and Extract Preparation

The samples were collected in May 2023 from four-year-old plants grown under field cultivation conditions at the cultivation base of Guangdong Xiaoyang Ecological Agriculture Co., Ltd. in Yunfu, China. The plant material was collected and authenticated by Dr. Ruifeng Ji as *N. speciosa*. A voucher specimen was deposited at Guangdong Pharmaceutical University under voucher No. 2023-015-143. No flowers were present at the time of collection. The collected aerial material consisted of leaves and stems, which were pooled and processed as a single composite sample; their relative proportions were not determined. The aerial parts of *N. speciosa* were dried, pulverised, and passed through a No. 4 sieve (approximately 65 mesh). The resulting powder was thoroughly mixed before subsampling. Powdered material (2.00 g) was extracted with methanol (40 mL) by ultrasonication at 40 °C for 30 min. The suspension was centrifuged at 1980× *g* for 15 min and the supernatant was collected. Solvent was removed under reduced pressure at 50–60 °C and 85–95 r/min, yielding 0.34 g of dry crude extract, corresponding to an extraction yield of 17.0% (*w*/*w*) relative to the initial dry plant material. The crude extract was obtained from a single preparative extraction; independent extraction replicates were not performed. For UHPLC-HRMS analysis, 1.0 mg of the extract was dissolved in 1 mL methanol and passed through a 0.22 μm organic-phase membrane filter (nylon). For antioxidant and enzyme inhibition assays, the extract was redissolved and diluted as described below.

### 2.2. UHPLC-Q-Orbitrap HRMS Analysis

Chromatographic separation was performed using a Vanquish Flex UHPLC system coupled to an Orbitrap Exploris 120 high-resolution mass spectrometer (Thermo Fisher Scientific, Waltham, MA, USA). An Xtimate UHPLC C18 column (2.1 × 100 mm, 1.8 μm; Welch Materials, Shanghai, China) was maintained at 35 °C. Mobile phase A was acetonitrile containing 0.1% formic acid and mobile phase B was water containing 0.1% formic acid. The gradient was: 0–5 min, 95–80% B; 5–8 min, 80–75% B; 8–20 min, 75–5% B; 20–22 min, 5% B; 22–25 min, 95% B. The flow rate was 0.30 mL/min and the injection volume was 2 μL.

Data were acquired separately in positive- and negative-ion electrospray ionisation modes after mass calibration. Spray voltages were +3.5 kV and −2.8 kV, respectively. The sheath-gas temperature was 350 °C, sheath-gas flow was 50 arbitrary units, auxiliary-gas flow was 8 arbitrary units, sweep-gas flow was 1 arbitrary unit, vaporiser temperature was 350 °C, and ion-transfer-tube temperature was 325 °C. Full-scan spectra were acquired over *m*/*z* 100–1500, and the first-order full scan resolution was 60,000, the second-order scan resolution was 15,000. The AGC target was set to Standard; the maximum injection time was set to Auto. The isolation window (*m*/*z*) was set to 1.5. Tandem mass spectra were acquired using normalised collision energy settings of 20, 40, and 60%.

### 2.3. Data Processing and Molecular Networking

Raw UHPLC-HRMS data were processed in Compound Discoverer 3.3 (Thermo Fisher Scientific). Features were extracted, elemental formulae were generated from accurate mass and isotope patterns, and MS/MS spectra were searched against mzCloud, mzVault, and an in-house library. Candidate annotations were retained when the mass error was within ±5 ppm and the reported library score exceeded 80.

For molecular networking, raw files were converted to mzML format using MSConvert and uploaded to the Global Natural Products Social (GNPS) molecular networking platform [23,24]. The cosine-score threshold was 0.50, at least six fragment ions were required for spectral matching, the TopK value was 10, and the maximum precursor-ion difference was 0.02 Da. Networks were visualised using Cytoscape 3.10.1.

### 2.4. Antioxidant Capacity

The extract (4 mg) was dissolved in methanol (2 mL) to obtain a 2 mg/mL stock solution and diluted as required. DPPH radical-scavenging, ABTS radical-cation-scavenging, ferric-reducing antioxidant power (FRAP), and Cu^2+^-reducing antioxidant capacity were determined in 96-well plates using commercial kits and Trolox calibration curves. Absorbance was recorded at 517, 734, 590, and 570 nm, respectively. Each measurement was performed in triplicate. Results were reported by the original dataset as Trolox-equivalent (TE) antioxidant capacity per gram of extract.

### 2.5. α-Glucosidase Inhibition Assay

*α*-Glucosidase (33.7 U/mg, catalogue no. S10050, Shanghai Yuanye Biotechnology Co., Ltd., Shanghai, China) inhibitory activity was determined using p-nitrophenyl-*α*-D-glucopyranoside (PNPG) as the substrate. *α*-Glucosidase and PNPG were prepared in phosphate-buffered saline (PBS; 0.1 M, pH 6.86). PBS (400 μL), *α*-glucosidase solution (400 μL, 0.25 U/mL), and extract solution (200 μL) were mixed and pre-incubated at 37 °C for 10 min. The reaction was initiated by adding PNPG solution (400 μL, 5 mmol/L) and incubated at 37 °C for 20 min. The reaction was terminated by adding 200 μL of 1.0 M Na_2_CO_3_ and mixing thoroughly. The final test concentration of the extract was 35, 25, 20, 15, 12.5, 10, 8, 5 μg/mL. An aliquot of the terminated reaction mixture (200 μL) was transferred to a 96-well plate, and the absorbance was measured at 405 nm. Acarbose was used as the reference inhibitor. In order to eliminate the influence of the colour and turbidity of the extract itself, each reaction hole was set up with a ‘sample blank control hole’ (without enzyme, the other components were the same), and the corresponding background absorbance was deducted when calculating the inhibition rate. Sample blanks without enzyme, enzyme controls without inhibitor, and reagent blanks without enzyme or inhibitor were prepared and treated identically. All concentrations are reported as the final concentrations in the reaction mixture.

Four conditions were included: sample with enzyme, sample blank without enzyme, enzyme control without inhibitor, and reagent blank without enzyme or inhibitor. Inhibition was calculated as: inhibition (%) = [1 − (A_sample_ − A_sample blank_)/(A_control_ − A_control blank_)] × 100. Concentration–response curves were fitted by nonlinear regression in GraphPad Prism, and IC_50_ values were calculated. The data were expressed as mean ± SD, from three independent biological replicates (*n* = 3), and each independent experiment had three replicates.

### 2.6. α-Amylase Inhibition Assay

*α*-Amylase (40–60 U/mg, SN: A769410, Shanghai MackLean Biochemical Technology Co., Ltd., Shanghai, China) inhibition was determined using the dinitrosalicylic acid (DNS) method. Soluble starch (1%, *w*/*v*) was prepared in PBS (0.1 M, pH 6.86). PBS (100 μL), *α*-amylase solution (100 μL, 2.5 U/mL), and extract solution (100 μL) were pre-incubated at 37 °C for 10 min. Starch solution (100 μL) was added to initiate the reaction, which proceeded at 37 °C for 10 min. DNS reagent (200 μL) was then added and the mixture was heated at 100 °C for 5 min. After cooling, 1 mL water was added, and 200 μL was transferred to a 96-well plate for absorbance measurement at 540 nm. The final test concentration of the extract was 1000, 500, 250, 125, 62.5, 50, 31.25, 12.5 μg/mL. Acarbose was tested in parallel. Blanks and inhibition calculations were defined as in Section 2.5. The data were expressed as mean ± SD, from three independent biological replicates (*n* = 3), and each independent experiment had three replicates.

### 2.7. Network Pharmacology Analysis

Canonical SMILES strings for the tentatively annotated constituents were retrieved using CAS Data/PubChem records. Potential protein targets were predicted using SwissTargetPrediction, retaining entries with probability > 0. Diabetes-associated targets were collected from GeneCards (https://www.genecards.org/), MalaCards (https://www.malacards.org/), OMIM (https://www.omim.org/), and the Therapeutic Target Database (https://ttd.idrblab.cn/) using the term “diabetes” (Website visit time December 2025). “Homo sapiens” was selected for all databases and enrichment tools. Compound-associated and disease-associated targets were intersected using Venny 2.1.0. Gene Ontology and Kyoto Encyclopedia of Genes and Genomes enrichment analyses were performed using Metascape (*p* < 0.05) [25,26]. A compound–target–pathway–disease network was constructed in Cytoscape 3.10.1, and compound centrality was assessed using betweenness centrality, closeness centrality, and degree centrality. All targets were uniformly corrected using UniProt ID. Compounds exceeding the median for all three measures were retained as network-prioritised candidates.

### 2.8. Molecular Docking

Eight network-prioritised compounds were docked against the sugar-beet *α*-glucosidase structural surrogate (PDB ID: 3W37, resolution 1.70 Å), human pancreatic *α*-amylase (PDB ID: 2QV4, resolution 1.97 Å), adenosine A2A receptor (*ADORA2A*; PDB ID: 3QAK), calcium/calmodulin-dependent protein kinase II *β* (*CAMK2B*; PDB ID: 3BHH), dopamine D2 receptor (*DRD2*; PDB ID: 9BS9), and endothelin receptor type A (*EDNRA*; PDB ID: 8XVJ) [27]. Protein structures were downloaded from the Protein Data Bank and prepared in PyMOL 3.0.0 by removing crystallographic water molecules, co-crystallised ligands, and other non-protein atoms. Catalytically essential calcium ions in PDB 2QV4 were retained during protein preparation. Hydrogen atoms and protonation states were assigned automatically using the default CB-Dock2 preparation protocol. Ligand structures were geometry-optimised and submitted together with the prepared protein structures to CB-Dock2 for automated blind docking [28]. For each ligand–protein pair, the highest-ranked docking pose and corresponding docking score reported by CB-Dock2 were retained, and representative binding poses were visualised in PyMOL.

### 2.9. Molecular Dynamics Simulation

In this study, Maestro 2023-2 software is used to simulate its dynamics. First, the Gly-353/Gly structure was introduced, the working path was changed, and the docking results were processed using the System Builder section in Desmond to generate a water box, in which the SPC solvent model and the OPLS4 position were used. The charge was neutralized by adding Na ions and Cl ions, and finally the minimum volume of the water box system was obtained by clicking on Minimize Volume. Secondly, the Minimization plate in Desmond was used to minimize the energy of the system. First, load the water box system, then set the minimization time to 500 ns, and click Run. Finally, the Molecular Dynamics plate in Desmond was used to simulate the dynamics of the system, and the simulation time was 100 ns. The Run interactions analysis was checked when the simulation job completed. After the simulation work is completed, use the Simulation Interaction Diagram section in Desmond to load the newly generated EAF file, and click on Generate Report in the upper right corner to generate a Data file, which contains root mean square deviation (RMSD), root mean square fluctuation (RMSF), and other images and data tables [19]. Ranging: Click the load trajectory in the project to import the dynamic trajectory, use the Measure plate in Desmond to select the target atom, automatically generate the distance between the atoms, click Measurements in the Plot module, and generate the dynamic distance image and data of the two atoms in the dynamic simulation. The binding energy between ligand and protein was calculated by MMGBSA method.

### 2.10. Statistical Analysis

Data are presented as mean ± the dispersion statistic reported in the original dataset. Concentration–response curves were fitted by nonlinear regression using GraphPad Prism 8.0.2. The fragmentation pathway of MS was drawn by Chemdraw 20.0.

## 3. Results and Discussion

### 3.1. Phytochemical Profile of the Aerial Part Extract

**Figure 1:**
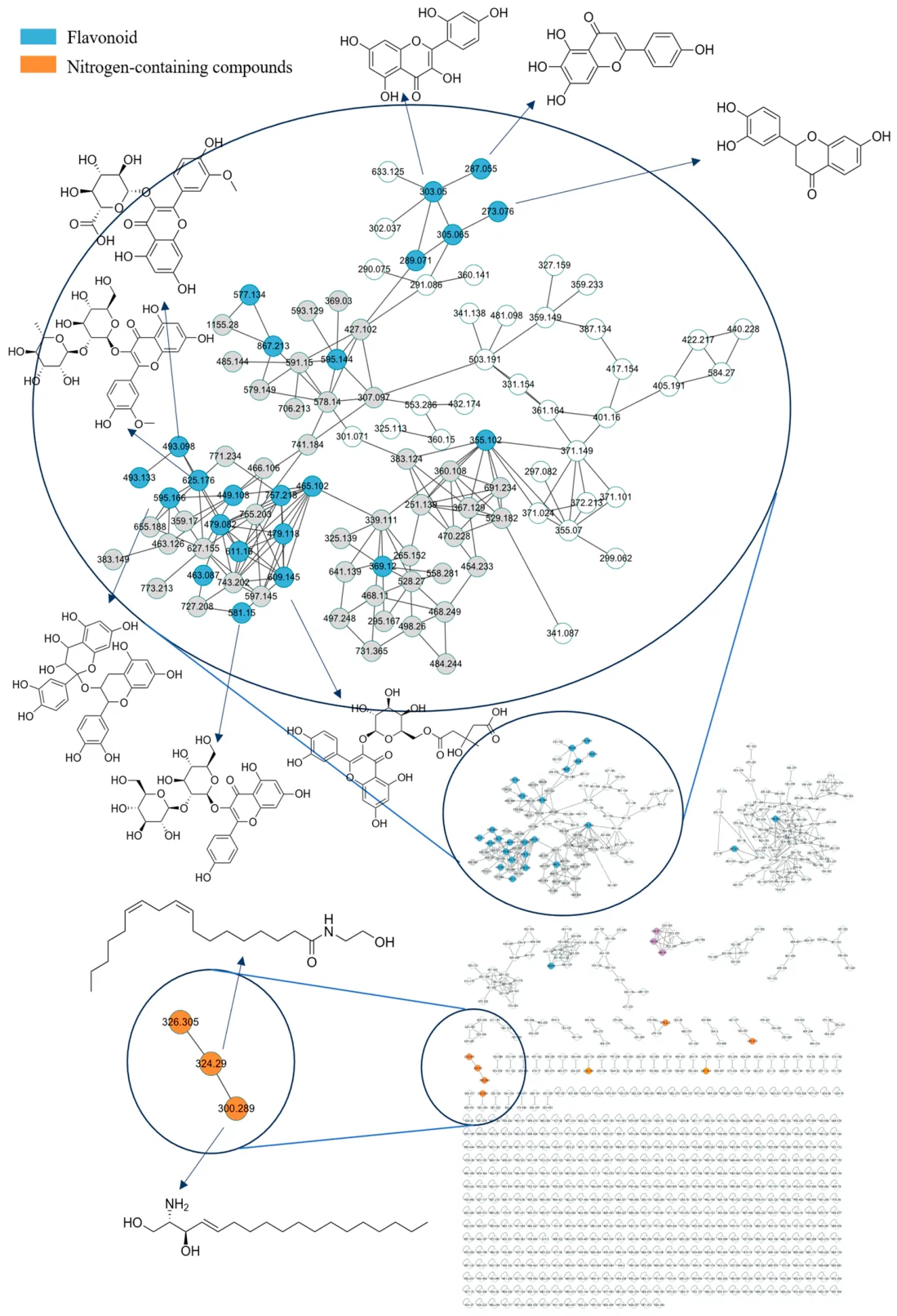
GNPS molecular network cluster of the aerial part of *N. speciosa* in positive ion mode.

The positive- and negative-ion base-peak chromatograms showed a chemically complex extract, with abundant polar features eluting during the first 8 min and additional lower-polarity features at later retention times (Supplementary Figure S1). The original LC-MS/MS data in positive ion mode was processed by the GNPS platform to construct a molecular network based on the similarity of MS/MS spectra (Figure 1). The network clusters compounds into different molecular clusters based on the consistency of secondary fragment ions, such as flavonoid clusters and nitrogen-containing compound clusters. Based on this clustering strategy, compounds with known structures can be used as anchors to infer the structure of unknown nodes in the cluster. The confirmation compound morin (*m*/*z* 303.0497, RT 6.662 min) with a matching score higher than 80 in the mass spectrometry database provides a reference for the annotation of subsequent associated nodes. By analysing the characteristic fragmentation rules of flavonoids, we locked the closely related nodes *m*/*z* 287.0550 and *m*/*z* 273.0790. Subsequently, combined with the accurate peak extraction of the original data and the manual verification of the corresponding secondary mass spectra one by one, the above nodes were finally tentatively annotated as scutellarein (*m*/*z* 287.0550, RT 5.531 min) and 2-(3,4-dihydroxyphenyl)-7-hydroxy-3,4-dihydro-2H-1-benzopyran-4-one (*m*/*z* 273.0757, RT 4.536 min), respectively. Similarly, based on the localization of the compound isorhamnetin **3**-glucuronide (*m*/*z* 493.0974, RT 8.005 min) in the network, we resolved the nodes *m*/*z* 625.1760 and *m*/*z* 595.1660. Subsequently, combined with the accurate peak extraction of the original data and the manual check of the corresponding secondary mass spectra one by one, the above nodes were finally tentatively annotated as the characteristic diglycoside flavonoid isorhamnetin 3-O-neohesperidoside (*m*/*z* 625.1762, RT 7.369 min) and the biflavonoid compound procyanidin with the same skeleton (*m*/*z* 595.1438, RT 14.978 min). Referring to the similar method, we also inferred the flavonoid glycoside compound quercetin **3**-O-[**6**’-O-(**3**-hydroxy-**3**-methylglutaroyl)-*β*-D-galactoside] (*m*/*z* 609.1449, RT 7.331 min), and the nitrogen-containing compound linoleoyl ethanolamide (*m*/*z* 324.2896, RT 18.133 min). It is worth noting that all the compounds derived from GNPS were subjected to secondary mass spectrometry comparison.

By combining Compound Discoverer 3.3 and GNPS, 96 tentative annotation results were generated (Supplementary Table S1). On the basis of the original grouping, these comprised 37 flavonoids and flavonoid glycosides, 19 nitrogen-containing compounds, 12 phenylpropanoid derivatives, 9 phenolic acids, 6 organic acids, 6 sugars or glycosides, and 7 other constituents.

**Figure 2:**
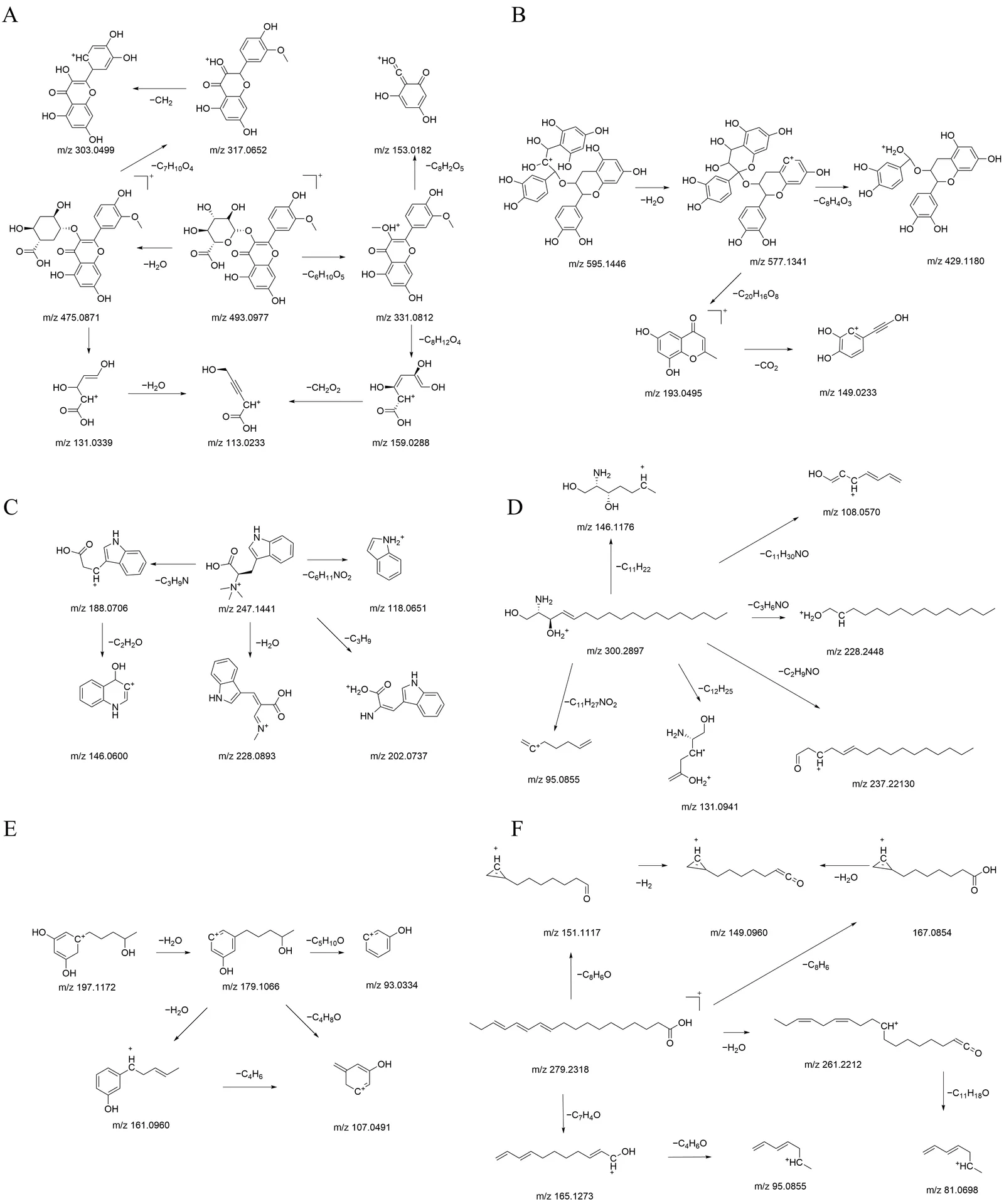
Possible fragmentation pathways of different compounds in *N. speciosa:* (**A**) isorhamnetin 3-glucuronide; (**B**) procyanidin; (**C**) (+)-hypaphorine; (**D**) sphingosine; (**E**) **5**-(**4**-hydroxypentyl)-**1**,**3**-benzenediol; (**F**) *α*-linolenic acid.

Flavonoids were the most diverse group and included quercetin, taxifolin, myricetin, morin, eriodictyol, scutellarein, rutin, isoquercitrin, quercitrin, trifolin, and multiple glucoside, rutinoside, galactoside, and glucuronide derivatives. Procyanidin dimers and oligomers were also tentatively assigned. In the positive ion mode, the compound 83 produces the quasi-molecular ion *m*/*z* 493.0977 [M+H]^+^, and its MS/MS fragmentation mainly produces characteristic fragments by glycosyl removal, dehydration, flavonol nucleus fragmentation and B-ring cracking. First, the quasi-molecular ion removes a molecule of H_2_O to form *m*/*z* 475.0871 [M+H‒H_2_O]^+^, and then further neutral loss of a residual sugar group, the formation of *m*/*z* 317.0652 [M+H‒H_2_O‒C_7_H_10_O_4_]^+^, and further removes CH_2_ to form *m*/*z* 303.0499. In the other fragmentation pathway, the parent ion directly neutrally loses a glucose residue (C_6_H_10_O_5_) to form *m*/*z* 331.0812 [M+H‒C_6_H_10_O_5_]^+^. Two characteristic ions *m*/*z* 153.0181 and *m*/*z* 159.0288 were generated by the further fragmentation of the C ring and B ring of flavonols, respectively. Among them, *m*/*z* 159.0288 further removed a molecule of formic acid (CH_2_O_2_) to generate *m*/*z* 113.0233. In addition, the dehydrated sugar fragment *m*/*z* 475.0871 was continuously cleaved by the sugar ring to form *m*/*z* 131.0339, and then a molecule of H_2_O was further removed to form *m*/*z* 113.0233. The above fragmentation behaviour shows that the compound has typical mass spectrometry fragmentation characteristics of flavanol glycosides. Combined with the database and literature comparison [29], it is inferred that the compound is isorhamnetin **3**-glucuronide, and its possible fragmentation pathway is shown in Figure 2A. Similarly, in the positive ion mode, compound 88 produces the quasi-molecular ion *m*/*z* 595.1446 [M+H]^+^, and its MS/MS fragmentation mainly produces characteristic fragments by dehydration, bond fragmentation between flavanol units and C-ring Retro-Diels-Alder (RDA) fragmentation. First, the quasi-molecular ion neutrally loses a molecule of H_2_O to form a fragment *m*/*z* 577.1341 [M+H‒H_2_O]^+^. Subsequently, the ion undergoes further RDA fragmentation, resulting in the neutral loss of C_8_H_4_O_3_ and the formation of the characteristic ion *m*/*z* 429.1180 [M+H‒H_2_O‒C_8_H_4_O_3_]^+^. In addition, *m*/*z* 577.1341 can also be cleaved by quinone methide between flavanol units, losing C_20_H_16_O_8_ to form monomer characteristic ion *m*/*z* 193.0495. The ion further removes a molecule of CO_2_ to form *m*/*z* 149.0233. The above fragmentation behaviour conforms to the typical mass spectrometry fragmentation rule of procyanidin dimer compounds. Combined with the database and literature comparison [30], it is inferred that the compound is procyanidin, and its possible fragmentation pathway is shown in Figure 2B. This profile is chemically consistent with the antioxidant and enzyme-inhibitory behaviour subsequently observed, because hydroxylated flavonoid scaffolds can participate in hydrogen bonding, π interactions, and hydrophobic contacts within enzyme-binding cavities.

The extract also contains the characteristics of betaine alkaloids (+)-hypnotine, trigonelline, adenosine, and the amino alcohol compound sphingosine, indicating that the aboveground part contains compounds that span multiple biosynthetic categories. For example, in the positive ion mode, compound 41 produces the quasi-molecular ion *m*/*z* 247.1441 [M+H]^+^, and its MS/MS fragmentation mainly produces characteristic fragments through quaternary ammonium side chain fragmentation, trimethylamine removal, side chain rearrangement, and indole parent nucleus fragmentation. First, the quasi-molecular ion neutrally loses a molecule of trimethylamine (C_3_H_9_N) to form *m*/*z* 188.0706 [M‒C_3_H_9_N]^+^. Subsequently, the ion underwent further side chain rearrangement and fragmentation to generate a stable hydroxyindolium ion *m*/*z* 146.0600. In the other fragmentation pathway, the quasi-molecular ion undergoes C*α*-C*β* bond fragmentation, and neutrally loses C_6_H_11_NO_2_ to form a typical indolinium characteristic ion *m*/*z* 118.0651. In addition, the quasi-molecular ions can undergo dehydration rearrangement to form *m*/*z* 228.0893, or through the removal of three methyl to form *m*/*z* 202.0737. The above fragmentation behaviour shows that the compound has typical mass spectrometry fragmentation characteristics of indole betaine alkaloids. Combined with the database and literature comparison [31], it is inferred that the compound is (+)-hypaphorine, and its possible fragmentation pathway is shown in Figure 2C. In addition, in the positive ion mode, the quasi-molecular ion *m*/*z* 300.2897 [M+H]^+^ formed by the compound is more prone to side chain breakage, removal of nitrogen-containing oxygen-containing groups and fragmentation of long-chain alkyl groups, resulting in a series of characteristic secondary fragments. The main fragmentation pathway is as follows: First, the nitrogen-containing oxygen-containing side chain in the molecule breaks, and *m*/*z* 228.2448 [M+H‒C_3_H_6_NO]^+^ fragments are formed after neutral loss of C_3_H_6_NO; or neutral loss of C_2_H_9_NO to form *m*/*z* 237.2213 [M+H‒C_2_H_9_NO]^+^ fragments. Secondly, the long-chain alkyl group was broken, C_11_H_22_ was neutrally lost, and nitrogen-containing and oxygen-containing fragments with *m*/*z* 146.1176 [M+H‒C_11_H_22_]^+^ were generated. C_12_H_25_ was neutrally lost through further fragmentation or another fragmentation pathway, forming *m*/*z* 131.0941 [M+H‒C_12_H_25_]^+^ fragments. The third step is the deep fragmentation of the molecular skeleton, which produces small molecule characteristic ions of *m*/*z* 108.0570 [M+H‒C_11_H_30_NO]^+^ and *m*/*z* 95.0855 [M+H‒C_11_H_27_NO_2_]^+^. Combined with the database and literature comparison [32], it is inferred that the compound is sphingosine, and its possible fragmentation pathway is shown in Figure 2D.

Phenolic acids and organic acids include gallic, protocatechuic, syringic, chlorogenic and *α*-linolenic acid. According to the characteristics of the secondary fragment, it was tentatively annotated and the mass spectrometry fragmentation rules were summarized. In the positive ion mode, the quasi-molecular ion *m*/*z* 197.1172 [M+H]^+^ produced by compound 53 is unstable, and it is prone to dehydration, side chain breakage and aromatic ring substituent fragmentation to generate a series of characteristic fragment ions. The main fragmentation pathway is as follows: first, the molecule neutrally loses a molecule of H_2_O to form *m*/*z* 179.1066 [M+H‒H_2_O]^+^ fragments; the side chain of the fragment is further broken, and C_5_H_10_O is neutrally lost, generating small fragments of aromatic rings with *m*/*z* 93.0334 [M+H‒H_2_O‒C_5_H_10_O]^+^. Second, the quasi-molecular ion peak continuously removes two molecules of H_2_O to form *m*/*z* 161.0960 [M+H‒2H_2_O]^+^ fragments; the fragment further neutrally loses C_4_H_6_, generating *m*/*z* 107.0491 [M+H‒2H_2_O‒C_4_H_6_]^+^ fragments. Third, *m*/*z* 179.1066 can also be broken by side chain rearrangement, neutral loss of C_4_H_8_O, directly forming *m*/*z* 107.0491 [M+H‒H_2_O‒C_4_H_8_O]^+^ fragments. Combined with the database and literature comparison [33], it is inferred that the compound is **5**-(**4**-hydroxypentyl)-**1**,**3**-benzenediol, and its possible fragmentation pathway is shown in Figure 2E. In the positive ion mode, compound 90 produces quasi-molecular ion *m*/*z* 279.2318 [M+H]^+^, which is more prone to fat chain breakage, dehydration and alkenyl chain rearrangement and fragmentation, forming multiple characteristic fragment ions. The main fragmentation pathway is as follows: First, the side chain of the parent ion is broken, and C_8_H_16_O is neutrally lost to form *m*/*z* 151.1117 [M+H‒C_8_H_16_O]^+^ fragments, which are further dehydrogenated to form *m*/*z* 149.0960 [M+H‒C_8_H_16_O‒H_2_]^+^. Second, the other side chain of the parent ion is broken, and C_7_H_14_O is neutrally lost to form *m*/*z* 165.1273 [M+H‒C_7_H_14_O]^+^ fragments, followed by neutral loss of C_4_H_6_O to form *m*/*z* 95.0855 [M+H‒C_7_H_14_O‒C_4_H_6_O]^+^ small molecule olefinic fragments. Third, the parent ion loses H_2_O neutrally through dehydration reaction to form *m*/*z* 261.2212 [M+H‒H_2_O]^+^ fragments, which are further cleaved and neutrally lose C_12_H_20_O to produce *m*/*z* 81.0698 [M+H‒H_2_O‒C_12_H_20_O]^+^ fragments. Fourth, the parent ion can also cause hydrocarbon chain rupture, neutral loss of C_8_H_16_, the formation of *m*/*z* 167.0854 [M+H‒C_8_H_16_]^+^ fragments, followed by further dehydration to generate *m*/*z* 149.0960 [M+H‒C_8_H_16_‒H_2_O]^+^. Combined with the database and literature comparison [34], it is inferred that the compound is *α*-linolenic acid, and its possible fragmentation pathway is shown in Figure 2F.

### 3.2. Antioxidant Capacity and Relevance to Diabetes-Associated Oxidative Stress

The aerial part extract showed measurable activity in all four chemical antioxidant assays (Table 1). The standard curves corresponding to the four methods are shown in Supplementary Figure S2. The reported TE values were 0.563 ± 0.010 for FRAP, 0.013 ± 0.001 for DPPH, 1.919 ± 0.035 for the Cu^2+^-reducing assay, and 0.190 ± 0.019 for ABTS. The strongest numerical response was obtained in the Cu^2+^-reducing assay, followed by FRAP, ABTS, and DPPH.

The detectable antioxidant response is consistent with the abundance of flavonoids, phenolic acids, procyanidins, and caffeoylquinic-acid derivatives. Phenolic hydroxyl groups can donate electrons or hydrogen atoms and can stabilise the resulting radicals through resonance. In the context of diabetes, such chemical reactivity is relevant because oxidative stress participates in *β*-cell dysfunction and vascular complications [35]. Nevertheless, DPPH, ABTS, FRAP, and Cu^2+^-reduction are cell-free assays. They do not account for absorption, metabolism, intracellular distribution, or endogenous antioxidant systems and therefore support only a complementary antioxidant property, not prevention or treatment of diabetic complications.

### 3.3. Inhibition of α-Glucosidase and α-Amylase

**Figure 3:**
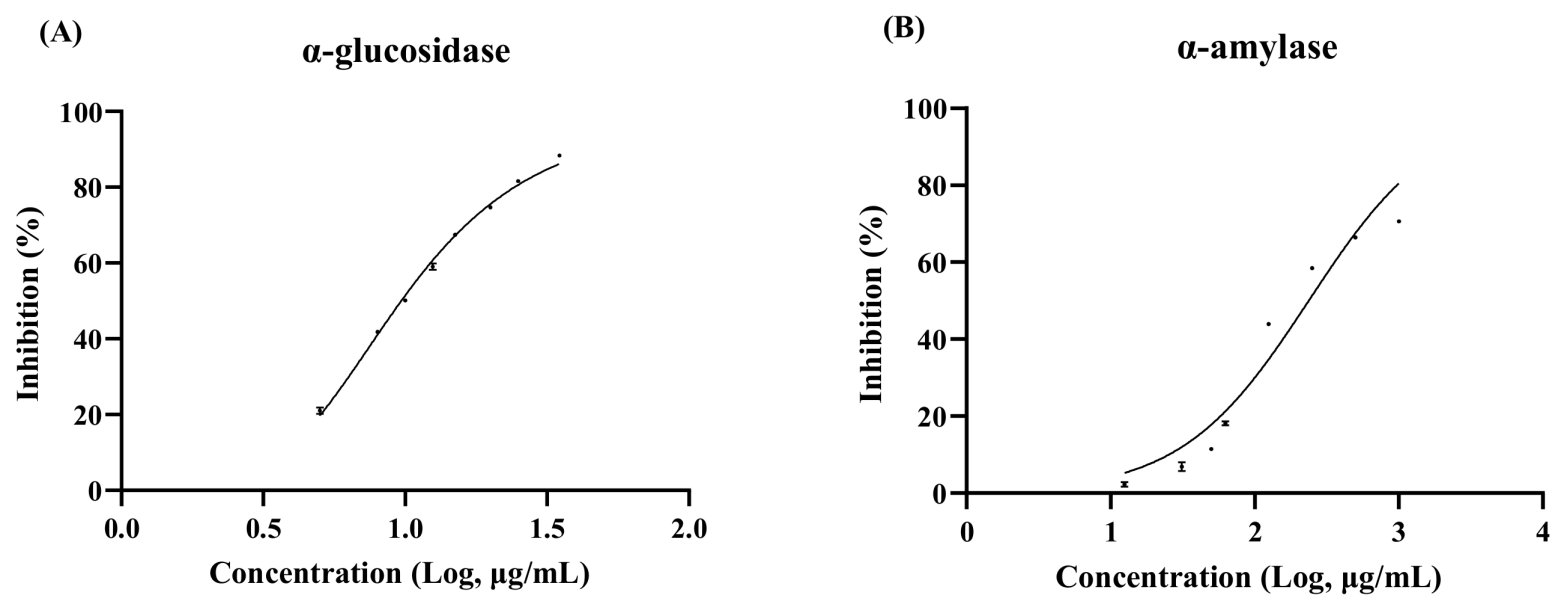
Concentration–response curves for inhibition of (**A**) *α*-glucosidase and (**B**) *α*-amylase by the aerial part extract and acarbose (*n* = 3, 95% CI).

The extract inhibited both carbohydrate-hydrolysing enzymes in a concentration-dependent manner (Figure 3). For *α*-glucosidase, inhibition reached 88.56 ± 0.14% at the highest reported concentration of 35 μg/mL, and the fitted value was 9.98 ± 0.02 μg/mL. Under the same assay conditions, acarbose gave an IC_50_ of 29.73 ± 0.04 μg/mL (Table 2). Thus, the extract showed an approximately threefold lower IC_50_ than acarbose in this particular enzyme system.

For *α*-amylase, inhibition increased across 0.125–2.000 mg/mL and reached 79.39 ± 0.67% at 2 mg/mL. The extract IC_50_ was 174.83 ± 1.31 μg/mL, whereas acarbose gave 39.46 ± 0.19 μg/mL; the extract was therefore approximately 4.4-fold less potent than acarbose against *α*-amylase. The ratio of *α*-amylase to *α*-glucosidase IC_50_ values for the extract was approximately 17.5, compared with 1.33 for acarbose, indicating a marked preference for *α*-glucosidase inhibition in the tested systems. A profile combining strong *α*-glucosidase inhibition with weaker *α*-amylase inhibition has been proposed as potentially useful for slowing terminal glucose release while avoiding excessive suppression of early starch digestion.

The phytochemical profile offers plausible candidates for the observed inhibition. Quercetin, taxifolin, myricetin, morin, and related flavonoids possess aromatic rings and multiple hydroxyl groups capable of interacting with catalytic or peripheral residues of glycosidases [36]. Caffeoylquinic acids, procyanidins, and other polyphenols may also contribute. A corresponding sample blank was included for each test condition to correct for extract-associated absorbance. Nevertheless, the DNS assay is a colorimetric endpoint method and cannot distinguish enzyme inhibition from reduced starch hydrolysis caused by nonspecific substrate binding or precipitation. Potential matrix effects of the crude extract on the high-temperature alkaline colour reaction also cannot be completely excluded. The observed *α*-amylase inhibition should therefore be interpreted as apparent activity under the established assay conditions. The underlying enzyme–inhibitor interactions and kinetic parameters require confirmation using more specific or complementary methods. These findings provide a basis for activity-guided isolation and subsequent identification of active constituents but do not establish the compounds responsible for the inhibition or the suitability of the extract as a food ingredient.

### 3.4. Network Pharmacology Prediction of Diabetes-Related Mechanisms

**Figure 4:**
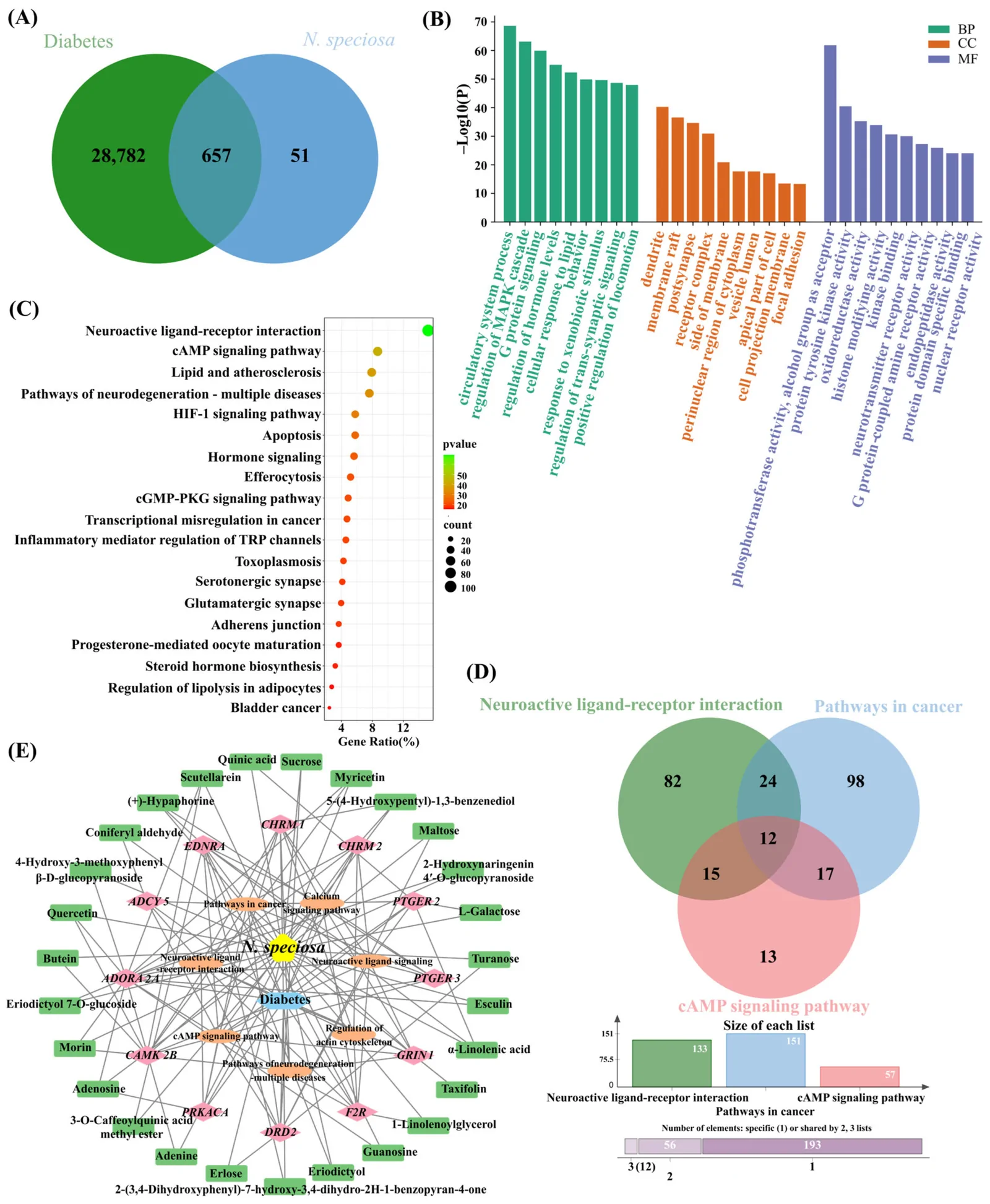
Network pharmacology workflow and results: (**A**) compound–disease target overlap; (**B**) GO enrichment; (**C**) KEGG enrichment; (**D**) overlap of targets in the three selected pathways; and (**E**) compound–target–pathway–disease network.

Target prediction for the 96 tentatively annotated constituents yielded 708 non-duplicate compound-associated targets. Searches of four disease databases using the broad term “diabetes” produced 29,439 disease-associated entries, and 657 targets overlapped between the two sets (Figure 4A). This large overlap reflects both the chemical diversity of the extract and the breadth of the disease query.

GO enrichment linked the overlapping targets to cellular responses to nitrogen compounds, circulatory-system processes, regulation of the MAPK cascade, membrane rafts, postsynaptic structures, phosphotransferase activity, protein-tyrosine kinase activity, and oxidoreductase activity. These terms are biologically broad but collectively point to signal transduction, redox regulation, and receptor-associated processes (Figure 4B). KEGG analysis highlighted pathways in cancer, neuroactive ligand–receptor interaction, and cAMP signalling (Figure 4C). “Pathways in cancer” should not be interpreted as a diabetes-specific mechanism; it is a composite pathway containing widely shared kinases, transcriptional regulators, survival signals, and metabolic nodes. Greater mechanistic relevance is provided by cAMP and calcium signalling, which participate in hormone secretion, receptor coupling, energy metabolism, and cellular responses to glucose [37,38].

Intersection of targets represented in the three highest-ranked pathways produced 12 central genes: *ADORA2A*, *CAMK2B*, *DRD2*, *EDNRA*, *ADCY5*, *PRKACA*, *PTGER2*, *F2R*, *PTGER3*, *CHRM2*, *CHRM1*, and *GRIN1* (Supplementary Table S2 and Figure 4D). *ADCY5* and *PRKACA* directly locate the network within the cAMP axis, whereas *CAMK2B* provides a connection to calcium-dependent signalling. *ADORA2A*, *DRD2*, prostaglandin receptors, muscarinic receptors, *EDNRA*, *F2R*, and *GRIN1* reflect a receptor-rich network capable of modulating second messengers and cellular excitability. These proteins are not established here as targets of the extract; their relevance derives from database-based prediction and topological overlap.

The compound–target–pathway–disease network contained 48 nodes and 137 edges (Figure 4E). Eight compounds exceeded the median values for betweenness, closeness, and degree centrality: 5-(4-hydroxypentyl)-1,3-benzenediol, (+)-hypaphorine, adenosine, *α*-linolenic acid, quinic acid, taxifolin, quercetin, and butein (Supplementary Table S3). The diversity of this set supports a multi-component hypothesis: flavonoids may provide polyphenol-driven enzyme and redox interactions; adenosine and hypaphorine may connect to receptor-related predictions; and quinic acid and *α*-linolenic acid may contribute different physicochemical and target profiles.

### 3.5. Molecular Docking Results

**Figure 5:**
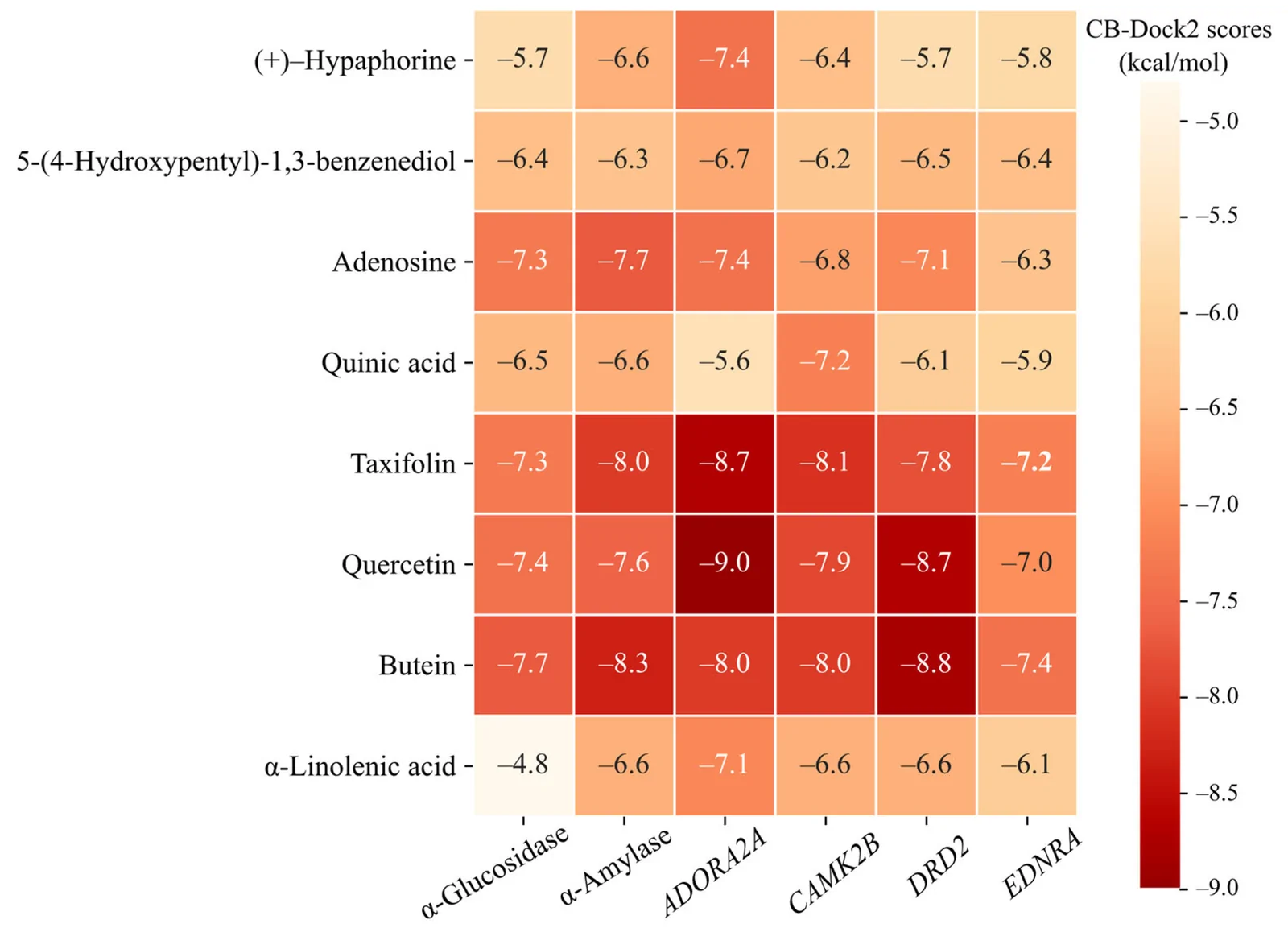
Heat map of CB-Dock2 scores (kcal/mol) for the eight prioritised compounds against six protein targets.

**Figure 6:**
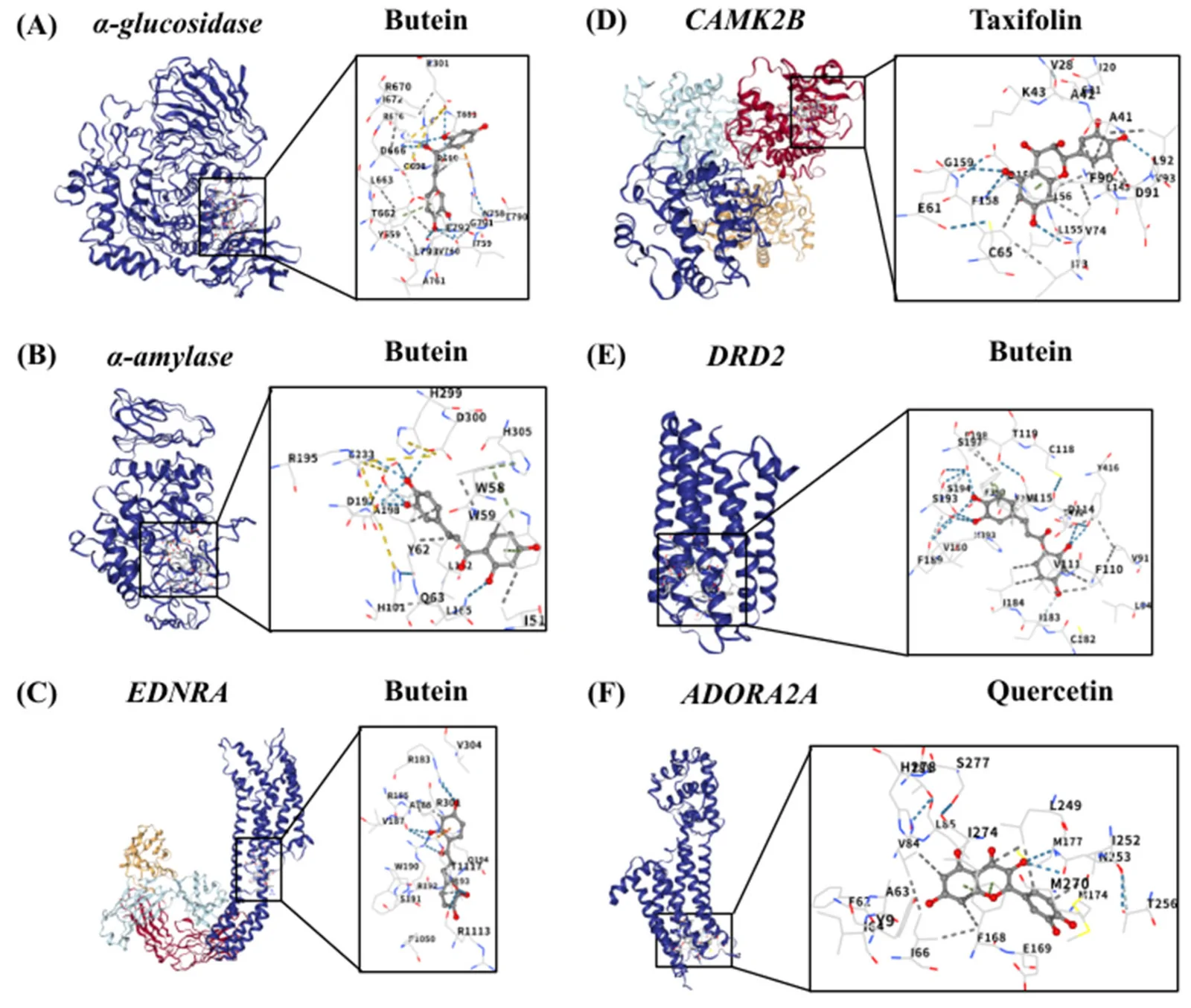
Representative docking poses: (**A**) butein–*α*-glucosidase; (**B**) butein–*α*-amylase; (**C**) butein–*EDNRA*; (**D**) taxifolin–*CAMK2B*; (**E**) butein–*DRD2*; and (**F**) quercetin–*ADORA2A*.

The eight network-prioritised candidates were docked against two carbohydrate-hydrolysing enzymes and four network-selected proteins (Figure 5). Scores ranged from −4.8 to −9.0 kcal/mol. Among the enzyme models, the feature assigned as butein produced the lowest reported scores for both *α*-glucosidase (−7.7 kcal/mol) and *α*-amylase (−8.3 kcal/mol), followed by quercetin, taxifolin, and adenosine. For the network-selected proteins, quercetin gave the lowest score with *ADORA2A* (−9.0 kcal/mol), taxifolin with *CAMK2B* (−8.1 kcal/mol), and butein with *DRD2* (−8.8 kcal/mol) and *EDNRA* (−7.4 kcal/mol) (Figure 6). These trends are structurally plausible for polyhydroxylated flavonoids, whose aromatic surfaces and hydroxyl groups can support multiple non-covalent contacts.

The docking provides a coherent structural extension for experimental observations. The relatively favourable scores of quercetin, taxifolin and butein characteristics for the enzyme model are consistent with the presence of polyphenol-rich extracts that preferentially inhibit *α*-glucosidase. The docking with *ADORA2A*, *CAMK2B*, *DRD2* and *EDNRA* also supports the geometric rationality of the selected edges in the pharmacological network. The preliminary results of molecular docking showed that butein had good binding conformation and binding energy advantages with the target protein. However, this method only reflects the local optimal binding state under static conditions, and it is difficult to fully simulate the dynamic stability and conformational adaptability of protein-ligand system in physiological environment. Therefore, molecular docking results alone are still not enough to reliably evaluate the true binding behaviour and persistence of ligands. Therefore, butein with a high docking score for several protein ligands can be used as a key compound for subsequent molecular dynamics simulation.

### 3.6. Molecular Dynamic Simulations

At the same time, although some studies have shown that butein has a potential regulatory role in metabolic disorders and inflammation-related pathways [39], its dynamic binding characteristics and possible new mechanisms in *α*-glucosidase and *α*-amylase and some protein systems still lack systematic research, especially in that the long-term conformational stability and key residue action modes have not been fully elucidated. Moreover, previous studies have revealed the reversible mixed inhibition mechanism of butein on *α*-glucosidase and pancreatic lipase by multi-spectral technology and molecular docking analysis, and elucidated the key role of butein in inducing enzyme microenvironment and conformational changes [40]. However, this study mainly relies on static docking and spectral experiments, and there is still a lack of systematic evaluation of the dynamic binding characteristics, long-term conformational stability and interaction evolution of key residues on the simulated time scale of the butein–enzyme complex. Acarbose is a commonly used *α*-glucosidase inhibitor in clinical practice. At the same time, it can competitively and reversibly inhibit pancreatic *α*-amylase and intestinal membrane-bound *α*-glucosidase. Therefore, it is often used as a positive control or reference drug in the study of glucose metabolism enzyme inhibition [41,42]. Therefore, acarbose was introduced as a positive control in this study. Based on this, in order to further reveal the potential mechanism of butein in this system and make up for the deficiency of molecular docking in dynamic information, this study further carried out molecular dynamics simulation analysis to systematically evaluate the structural stability and interaction evolution process of its complex.

**Figure 7:**
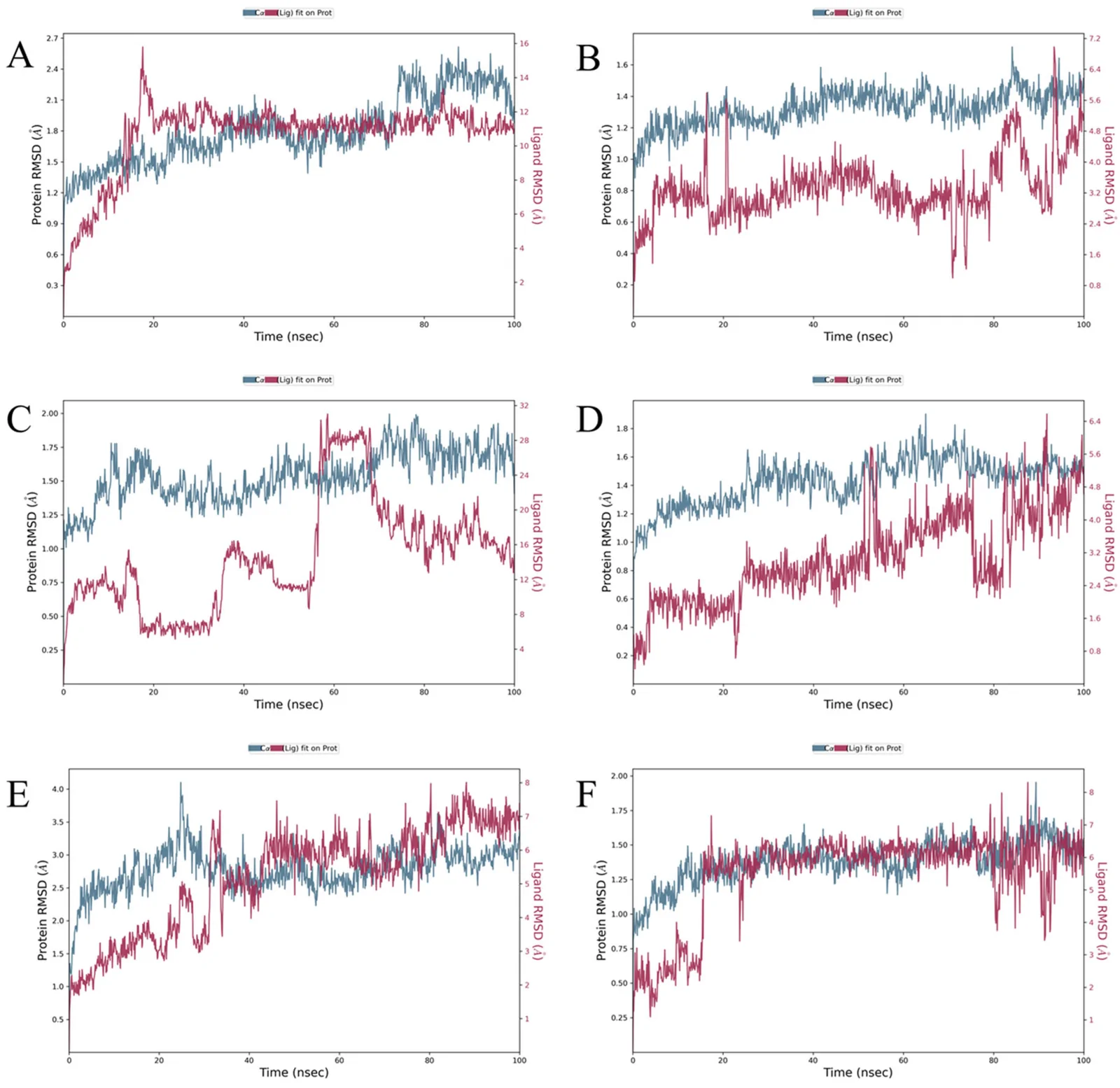
RMSD analysis of six systems: (**A**) acarbose–*α*-amylase; (**B**) butein–*α*-amylase; (**C**) acarbose–*α*-glucosidase; (**D**) butein–*α*-glucosidase; (**E**) acarbose–*DRD2*; (**F**) butein–*DRD2*.

RMSD was used to assess the conformational stability of the protein–ligand complexes during molecular dynamics simulations [43]. In the *α*-glucosidase complexes, the protein backbone RMSD stabilised at approximately 1.4–1.5 Å for both ligand-bound systems, indicating limited deviation from the initial protein structure. A similar pattern was observed for *α*-amylase, with backbone RMSD values remaining within approximately 1.3–1.8 Å. The *DRD2* complexes showed a different pattern. The protein backbone RMSD reached approximately 2.8 Å in the acarbose-bound system and 1.3 Å in the butein-bound system. The acarbose-bound *DRD2* complex therefore exhibited a greater deviation from its initial receptor conformation, although the trajectory remained within a bounded fluctuation range (Figure 7). Because a single ligand RMSD value cannot distinguish displacement within the binding pocket from changes in the ligand conformation, ligand behaviour was evaluated using two complementary measures. The binding-site-relative RMSD, calculated after alignment to the protein, reflects changes in the ligand position and orientation within the pocket, whereas the internal RMSD reflects conformational changes relative to the initial bound structure. In the *α*-glucosidase complex, the binding-site-relative RMSD of butein remained at approximately 3.1 Å, while its internal RMSD was approximately 0.65 Å, indicating limited internal deformation and a comparatively stable position within the binding pocket. Acarbose showed a substantially higher binding-site-relative RMSD of approximately 14.6 Å, including a transient peak of approximately 31 Å, consistent with marked displacement or reorientation from its initial binding pose. A similar difference was observed in the α-amylase complexes, with binding-site-relative RMSD values of approximately 3.3 Å for butein and 10.6 Å for acarbose. In the *DRD2* complexes, both ligands showed moderate deviations of approximately 5 Å from their initial binding poses, suggesting some positional reorganisation within the receptor-binding environment.

**Figure 8:**
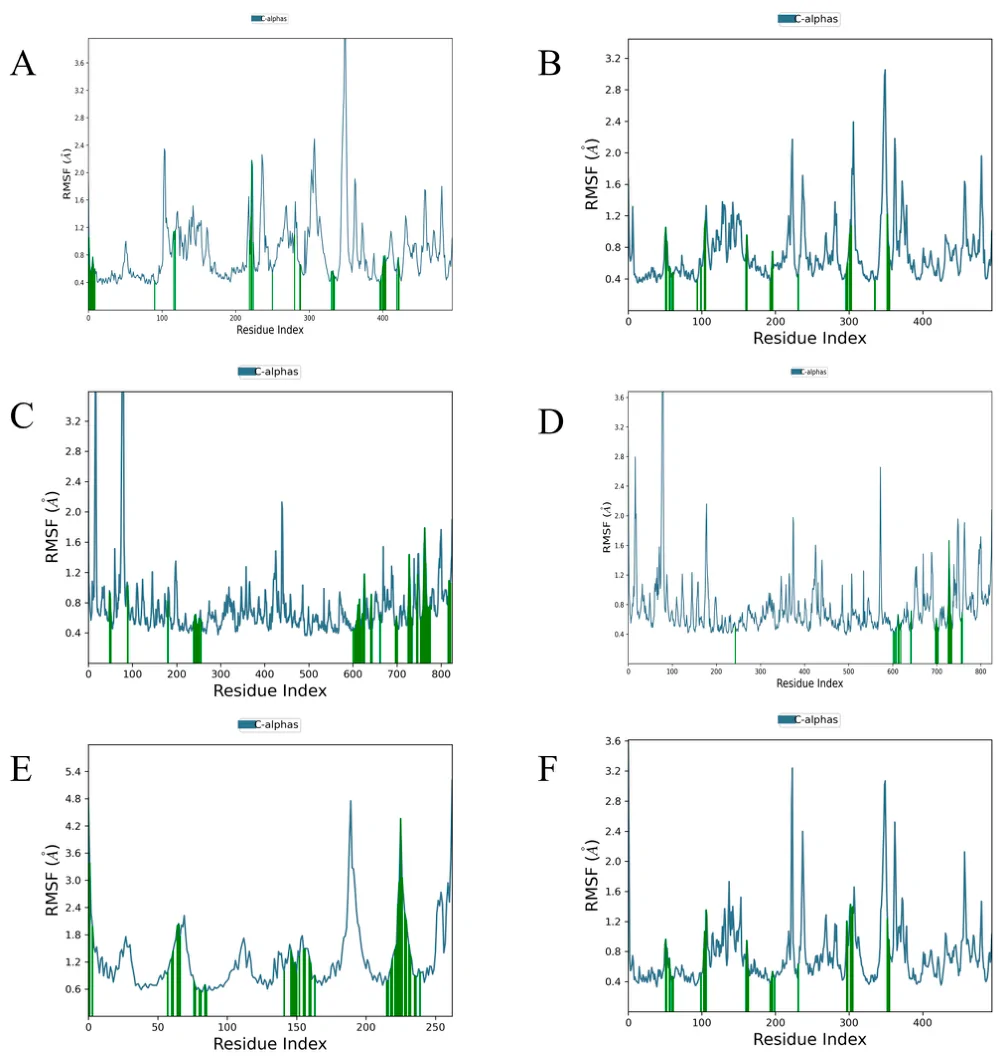
RMSF distribution of protein residues in six complex systems: (**A**) acarbose–*α*-amylase; (**B**) butein–*α*-amylase; (**C**) acarbose–*α*-glucosidase; (**D**) butein–*α*-glucosidase; (**E**) acarbose–*DRD2*; (**F**) butein–*DRD2* (Green: RMSF of the residues in the ligand binding pocket; blue: RMSF of the residue).

RMSF is often used to analyse the local flexibility at the residue level in molecular dynamics simulation, and a higher peak value usually indicates that the corresponding region has strong conformational fluctuations [43,44]. In the analysis of protein flexibility, the overall average RMSF in the *α*-glucosidase system is about 0.74 Å (butein system) and 0.75 Å (acarbose system), and the *DRD2* system is about 1.28 Å, indicating that the overall flexibility of the protein under the action of different ligands is small and the stability of the system structure is high (Figure 8). It is worth noting that obvious local flexibility peaks (RMSF > 7Å) were observed in the PRO116-PRO118 region, which belongs to the natural high-flexibility ring region, mainly reflecting the inherent dynamic characteristics of the protein, rather than ligand-induced instability. In spite of the existence of local high fluctuation regions, butein still maintained a lower ligand RMSD in the glycosidase system, indicating that its binding mode had good adaptability to the local flexibility of the protein.

**Figure 9:**
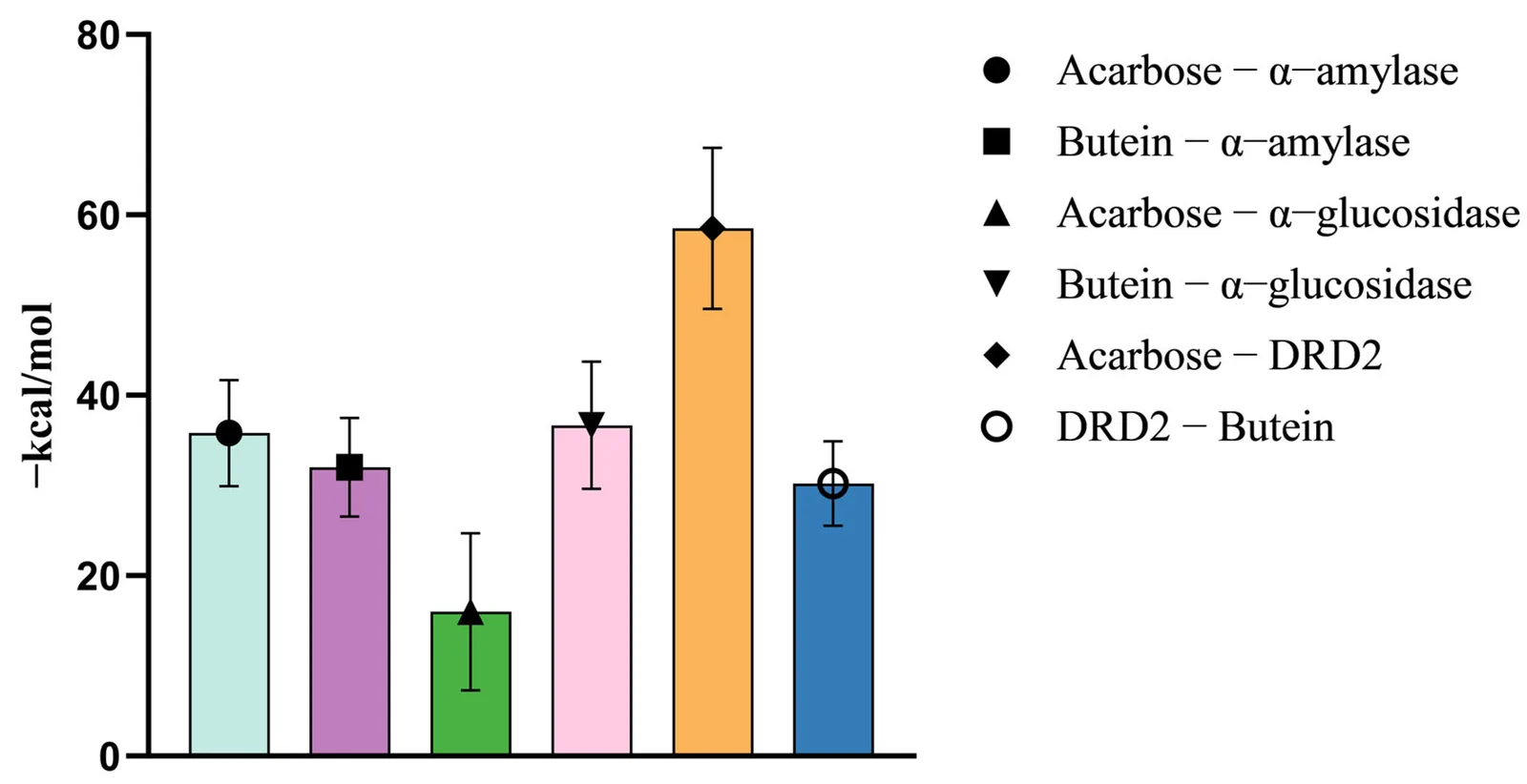
Comparison of MM/GBSA binding free energies of six protein–ligand complexes.

In terms of binding free energy, the binding free energy of butein in the *α*-glucosidase system is −36.68 kcal/mol, while that of acarbose is −16.00 kcal/mol, and the difference between the two is more than 20 kcal/mol, indicating that butein has a more favourable thermodynamic binding trend in this system (Figure 9). In the *α*-amylase system, acarbose was slightly better than butein (−35.81 kcal/mol vs. −32.03 kcal/mol), while acarbose showed the strongest binding free energy (−58.52 kcal/mol) in the *DRD2* system. It should be pointed out that the binding free energy reflects the thermodynamic preference, while the RMSD reflects the dynamic stability. MM/GBSA estimates relative binding trends rather than experimental binding affinity. The two do not need to be completely consistent, so it is a normal phenomenon that there is a certain deviation in some systems. Related MM/GBSA methodology reviews have also pointed out that this type of end-point free energy calculation is suitable for binding trend judgment [45,46].

RMSD describes the overall structural behaviour of a system but does not identify the interactions that maintain a ligand within the binding pocket. The occupancies of non-covalent interactions were therefore examined throughout the trajectories (Supplementary Table S5 and Figure S3). In the *α*-glucosidase complex, acarbose interacted mainly through hydrogen bonds and water bridges with charged or polar residues, including Glu, Asp, and Arg. Although numerous contacts were observed, no individual hydrogen bond exceeded 30% occupancy, and the highest occupancy was approximately 21%. Water-mediated interactions were more persistent; for example, the water bridge with Glu792 showed an occupancy of approximately 33%. Hydrophobic and π-related interactions were almost absent. The limited persistence of direct contacts and the greater dependence on water-mediated interactions may be associated with the observed variability in the acarbose binding pose.

Butein displayed a different interaction pattern. In addition to hydrogen bonding, it formed π–π stacking interactions with Tyr residues and π–cation interactions with Arg residues. In the *α*-glucosidase complex, persistent hydrogen bonds were observed with Ile759 (96%), Glu792 (59%), and Arg676 (44%), together with π–π stacking involving Tyr659 (37%) and a π–cation interaction with Arg699 (29%). This combination of interactions may restrict ligand movement and contribute to the comparatively stable binding pose of butein. Flavonoid inhibition of *α*-glucosidase and *α*-amylase is influenced by the aromatic scaffold and hydroxyl substituents that mediate interactions with residues in the binding pocket [3]. The interactions observed for butein are consistent with these structural features and may help explain its relatively low binding-site-relative RMSD in the two enzyme complexes.

The residue-by-interaction fingerprints further illustrate the differences between the two ligands (Supplementary Figure S4). Butein formed a diverse set of contacts, including hydrogen bonds, π–π stacking, π–cation interactions, hydrophobic contacts, and water bridges. The acarbose interaction profile was dominated by hydrogen bonds and water bridges, with little contribution from π–related interactions. These fingerprints were consistent with the interaction-occupancy analysis and provided a more detailed view of ligand behaviour at the binding interfaces.

The radius of gyration (Rg) describes the spatial distribution of ligand atoms around their centre of mass and was used to assess changes in ligand compactness. For a given ligand, a narrow Rg distribution indicates limited conformational variation, whereas a wider distribution suggests greater changes in molecular shape. Absolute Rg values should not, however, be compared directly between chemically different ligands because they also depend on molecular size and architecture. Across the six systems, the Rg of butein in the *α*-glucosidase complex remained at approximately 4.28 Å (range: 4.01–4.47 Å), with a standard deviation of approximately 0.07 Å. Its Rg in the *α*-amylase complex was approximately 4.35 Å, indicating limited variation in molecular compactness during the trajectory. Acarbose showed a wider Rg distribution in the *α*-glucosidase complex, with a mean of approximately 5.46 Å and a range of 4.96–6.19 Å. This variation indicates greater internal conformational flexibility, while its high binding-site-relative RMSD separately reflects displacement or reorientation within the binding pocket. The mean Rg values of acarbose were approximately 5.50 Å in the *α*-amylase complex and 5.89 Å in the *DRD2* complex. The *DRD2* trajectory showed the widest range, from 5.39 to 6.23 Å. Overall, the narrower Rg fluctuations of butein indicate more limited changes in its internal conformation. Together with the RMSD and interaction analyses, these results describe distinct binding and conformational behaviours for the two ligands across the simulated protein systems.

Each protein–ligand complex was represented by a single 100 ns trajectory, without independent replicate simulations. The MD and MM/GBSA results should therefore be regarded as preliminary supporting evidence rather than a demonstration of reproducible differences between the ligand systems. Multiple independently initiated simulations will be required to assess the robustness of these observations. This limited conformational sampling represents an important limitation of the present computational analysis.

## 4. Conclusions

This study demonstrates that the underutilised aerial parts of *N. speciosa*, which are frequently discarded despite the established utilisation of the roots, contain a chemically diverse phytochemical profile and measurable functional activities. UHPLC-Q-Orbitrap HRMS combined with GNPS molecular networking and representative MS/MS fragmentation analysis enabled the tentative annotation of 96 constituents, with flavonoids and flavonoid glycosides representing the most diverse chemical group. The aerial-part extract inhibited both *α*-glucosidase and *α*-amylase in a concentration-dependent manner and showed a pronounced preference for *α*-glucosidase under the present experimental conditions, together with measurable chemical antioxidant capacity.

Network pharmacology provided hypothesis-generating associations with cAMP-, calcium-, and receptor-related signalling, while molecular docking prioritised butein as a candidate constituent with favourable predicted interactions with both carbohydrate-hydrolysing enzymes. Molecular dynamics and MM/GBSA analyses further supported the predicted compatibility of butein with the enzyme-binding regions, but these computational results do not establish direct target engagement or confirm the contribution of butein to the activity of the crude extract. Because the extract was prepared only once, between-extraction variability and process reproducibility could not be assessed. From a valorisation perspective, the findings demonstrate the phytochemical potential and preliminary in vitro bioactivity of *N. speciosa* aerial biomass. However, they do not establish its suitability as a food or functional ingredient. Isolation of prioritised constituents, enzyme-kinetic validation, and assessment of safety, bioaccessibility, stability, and performance in relevant food matrices will be required before practical food-related applications can be considered.

## Figures and Tables

**Table 1:** Antioxidant capacity of the aerial part extract.

Assay	Antioxidant Capacity
FRAP	0.563 ± 0.010
DPPH	0.013 ± 0.001
Cu^2+^ reduction	1.919 ± 0.035
ABTS	0.190 ± 0.019

The values are reported as mean ± SD. The unit was mmol TE/g extract.

**Table 2:** Inhibitory activity against *α*-glucosidase and *α*-amylase.

Enzyme	Extract IC_50_ (μg/mL)	Acarbose IC_50_ (μg/mL)
*α*-Glucosidase	9.98 ± 0.02	29.73 ± 0.04
*α*-Amylase	174.83 ± 1.31	39.46 ± 0.19

Three groups were measured in parallel in each experiment. The values are reported as mean ± dispersion.

## Data Availability

The original contributions presented in this study are included in the article/Supplementary Materials. Further inquiries can be directed to the corresponding authors.

## Supplementary Materials

The following supporting information can be downloaded at https://www.mdpi.com/article/10.3390/foods15173167/s1, Figure S1: Positive- and negative-ion base-peak chromatograms of the methanolic extract of the aerial parts of *N. speciosa*; Figure S2: Standard curves of four antioxidant methods; Figure S3: PL-Contacts Histogram; Figure S4: LP-Contacts_2d-Summary; Figure S5: L-Properties; Table S1: UHPLC-Q-Orbitrap HRMS tentative annotation results of the aerial parts of *N. speciosa*; Table S2: Twelve central targets obtained from the pathway-overlap analysis; Table S3: Network centrality of the eight prioritised compounds; Table S4: RMSD values and radius of gyration (Rg) of the ligands in six simulated systems; Table S5: Representative protein–ligand contacts and their occupancies (%) over the trajectory in six simulated systems.
