# Supplementary material for: Phytochemical Profiling and α-Glucosidase and α-Amylase Inhibitory Activities of Underutilised Nanhaia speciosa Aerial Biomass
Source: Foods. 2026 Sep 7;15(17):3167. doi: 10.3390/foods15173167 (PMC13565697; doi:10.3390/foods15173167)
Supplement: Supplementary file 1 [file foods-15-03167-s001.zip › foods-4521150-supplementary.pdf]

Supplementary Materials

List of Supporting Information

Figure S1. Positive- and negative-ion base-peak chromatograms of the methanolic extract of the aerial parts of *N. speciosa*. 1

Figure S2. Standard Curves of Four Antioxidant Methods. 2

Figure S3. PL-Contacts Histogram. 3

Figure S4. LP-Contacts_2d-Summary. 4

Figure S5. L-Properties. 5

Table S1. The UHPLC-Q-Orbitrap HRMS tentative annotation results results of the aerial parts of *N. speciosa* 6

Table S2. Twelve central targets obtained from the pathway-overlap analysis. 11

Table S3. Network centrality of the eight prioritised compounds. 12

Table S4. RMSD values and radius of gyration (Rg) of the ligands in six simulated systems. 13

Table S5. Representative protein–ligand contacts and their occupancies (%) over the trajectory in six simulated systems. 14

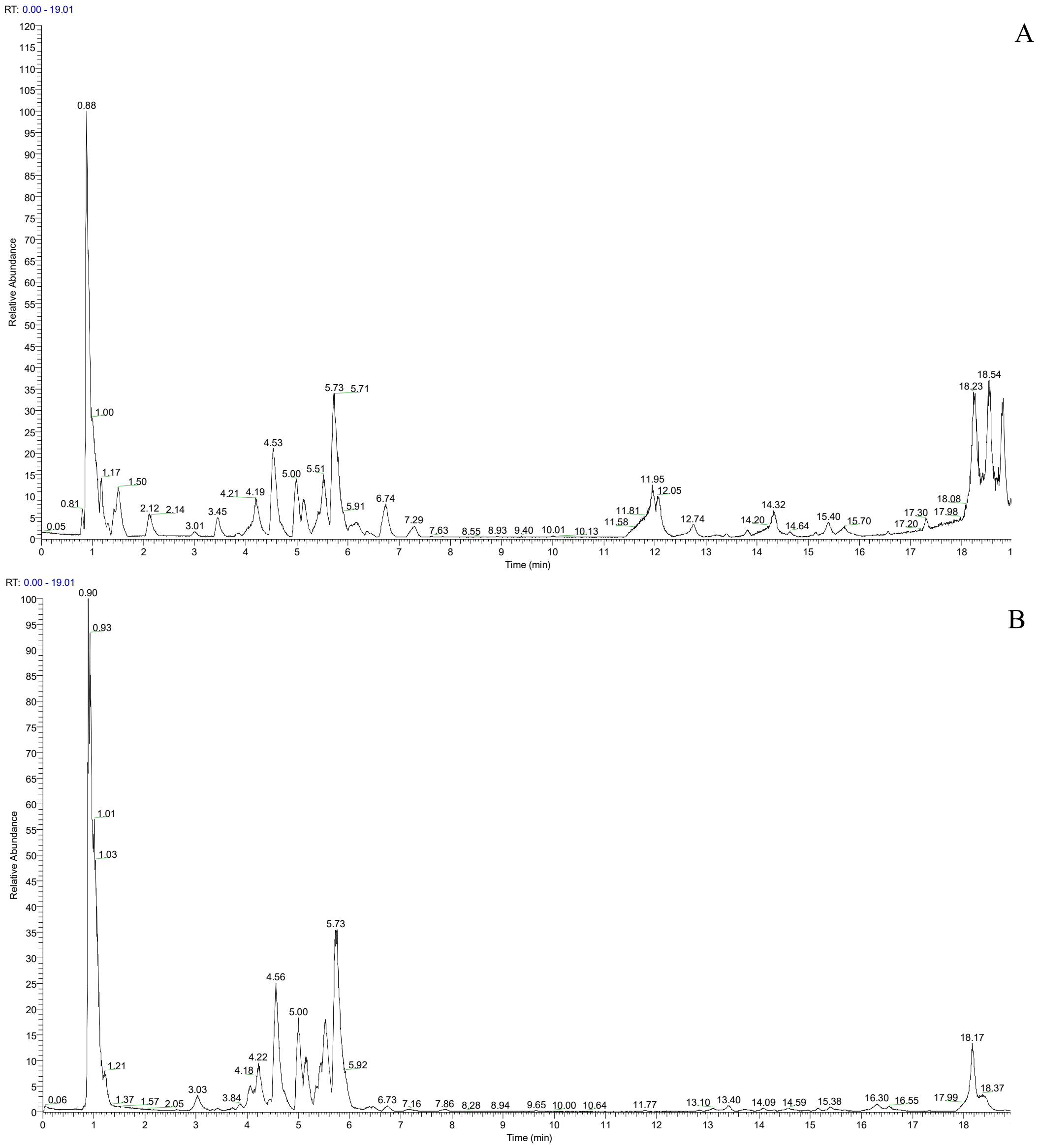

Figure S1. Positive- and negative-ion base-peak chromatograms of the methanolic extract of the aerial parts of *N. speciosa*.
(A: Positive ion mode; B: Negative ion mode)

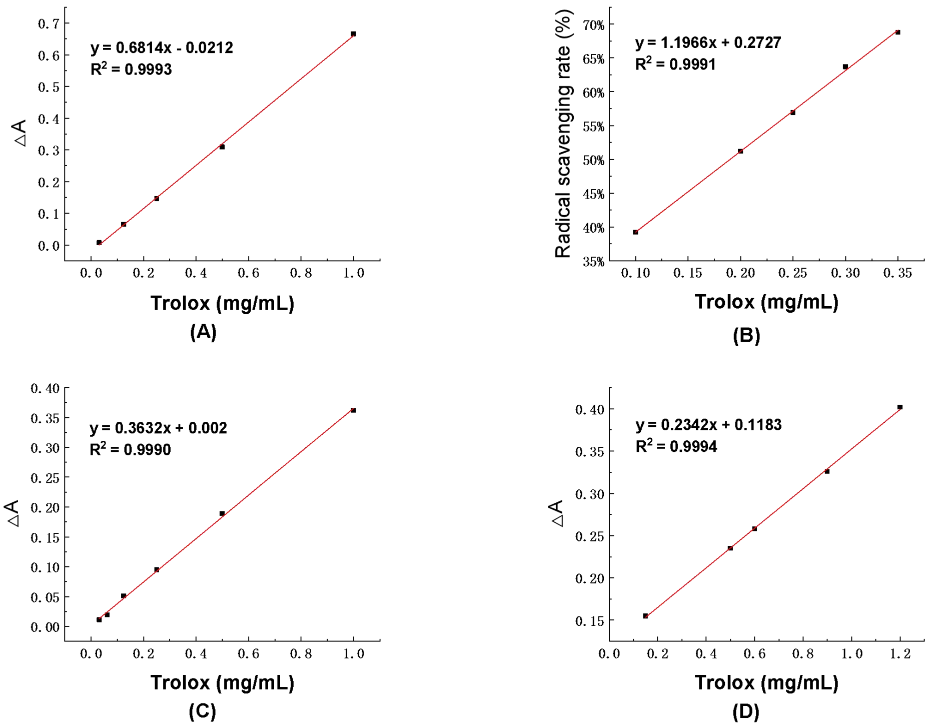

Figure S2. Standard Curves of Four Antioxidant Methods.
(A: FRAP; B: DPPH; C: Cu^{2+}; D: ABTS)

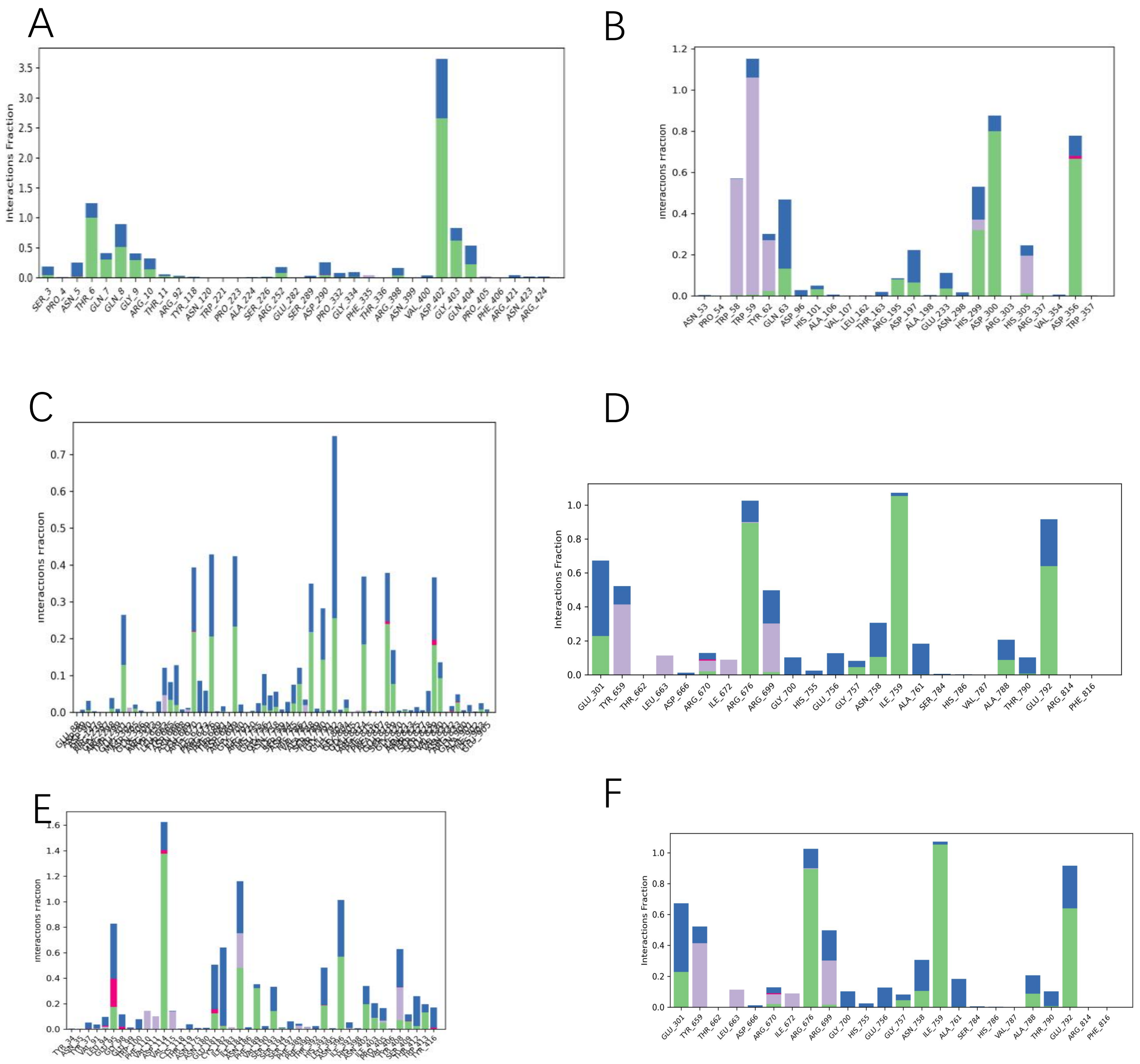

Figure S3. PL-Contacts Histogram.

(A) acarbose–α-amylase; (B) butein–α-amylase; (C) acarbose–α-glucosidase; (D) butein–α-glucosidase; (E) acarbose–DRD2; (F) butein–DRD2.

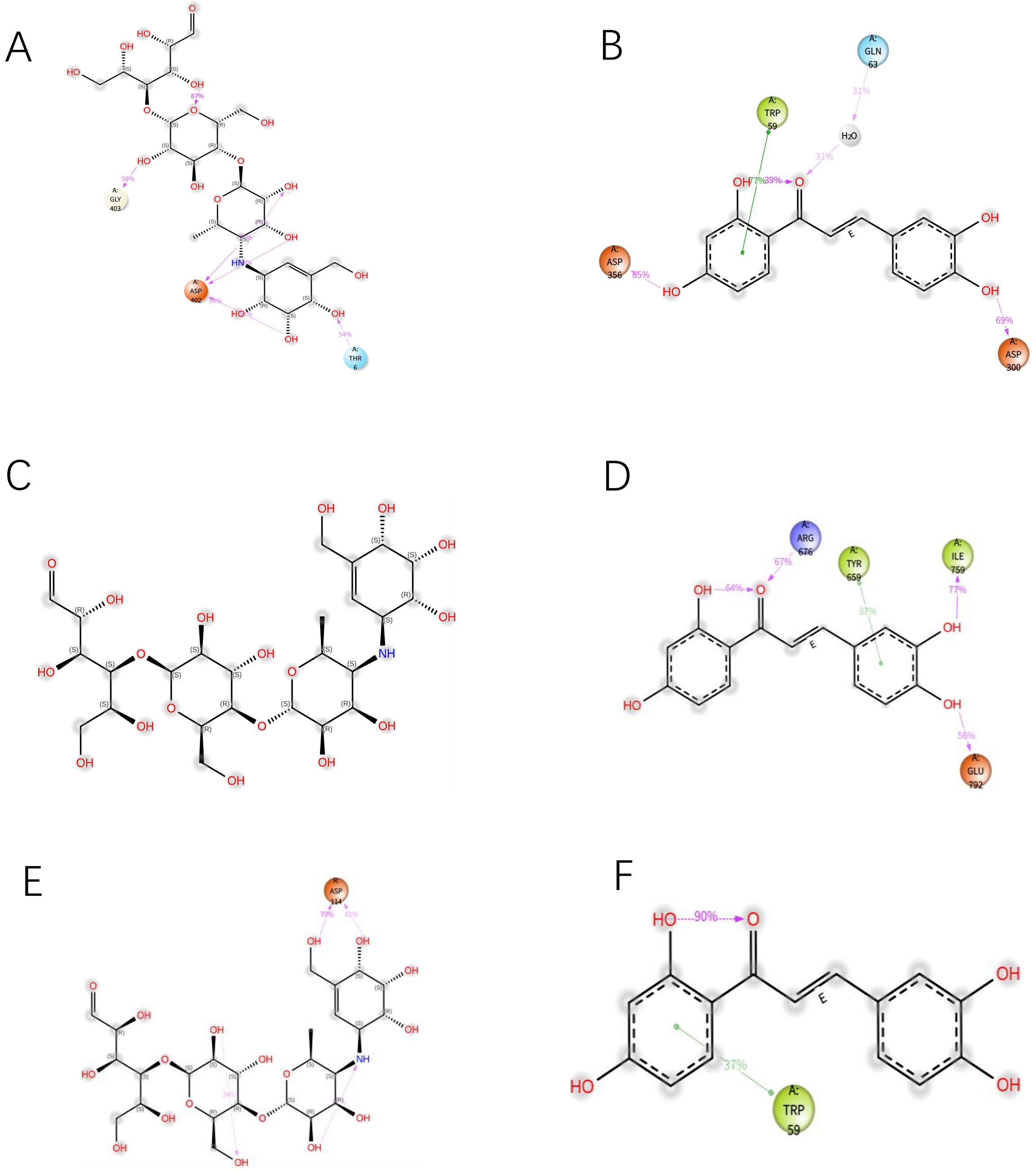

Figure S4. LP-Contacts_2d-Summary.

(A) acarbose–α-amylase; (B) butein–α-amylase; (C) acarbose–α-glucosidase; (D) butein–α-glucosidase; (E) acarbose–DRD2; (F) butein–DRD2.

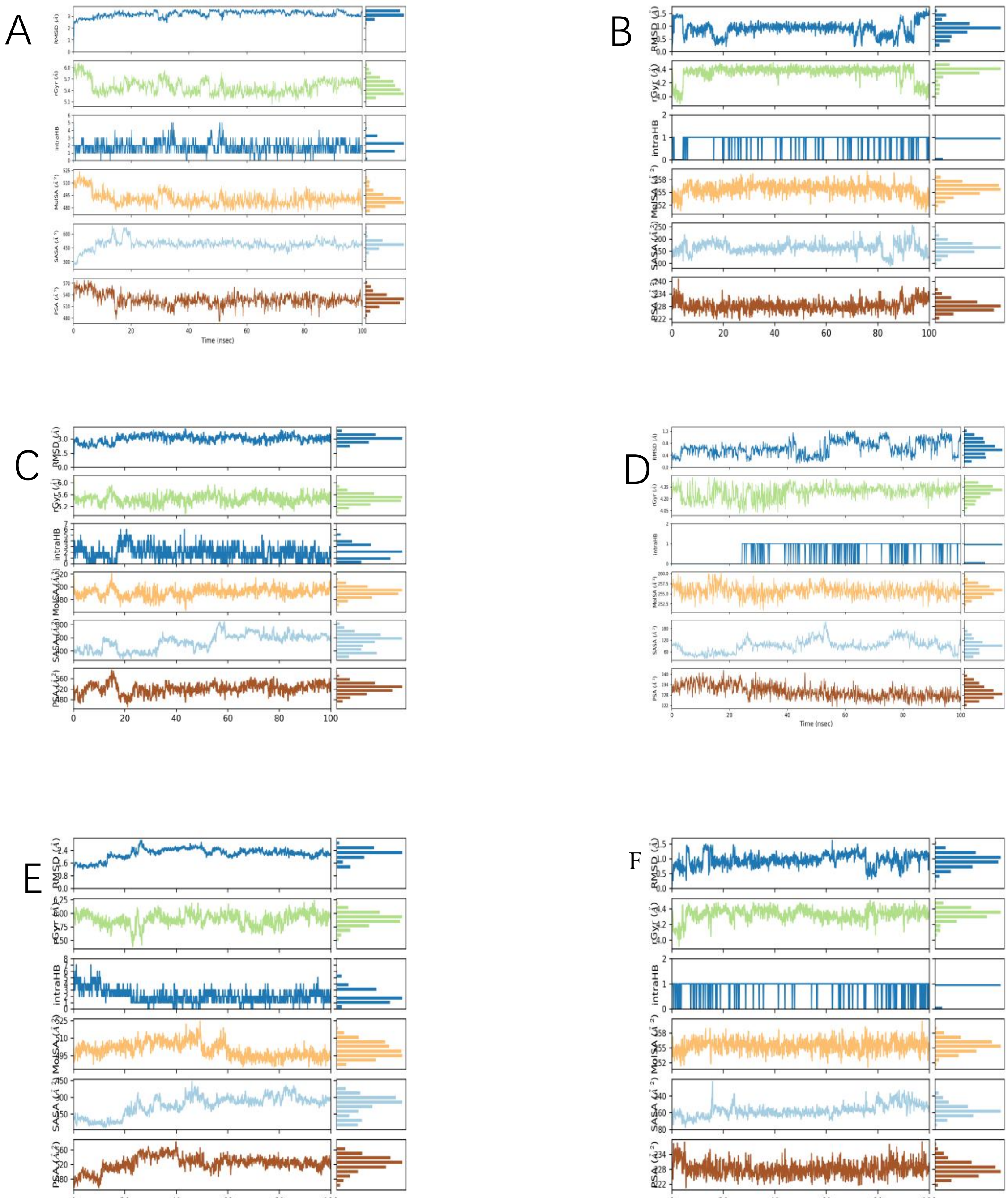

Figure S5.L-Properties.

(A) acarbose–α-amylase; (B) butein–α-amylase; (C) acarbose–α-glucosidase; (D) butein–α-glucosidase; (E) acarbose–DRD2; (F) butein–DRD2.

Table S1. The UHPLC-Q-Orbitrap HRMS tentative annotation results results of the aerial parts of *N. speciosa*

NO	RT /min	Compound	Formula	Mass deviation /ppm	m/z	Ion addition mode	Confidence	Structure type
1	0.924	erlose	$C_{18}H_{32}O_{16}$	0.21	503.1619	$[M–H]^–$	II	Sugars and glycosides
2	0.927	L–galactose	$C_6H_{12}O_6$	–0.45	179.0560	$[M–H]^–$	II	Sugars and glycosides
3	1.056	L–malic acid	$C_4H_6O_5$	–1.57	133.0140	$[M–H]^–$	II	Organic acids
4	1.254	citric acid	$C_6H_8O_7$	–0.98	191.0196	$[M–H]^–$	I	Organic acids
5	1.349	turanose	$C_{12}H_{22}O_{11}$	–0.57	342.1160	$[M+H]^+$	II	Sugars and glycosides
6	1.351	maltose	$C_{12}H_{22}O_{11}$	–0.57	381.0791	$[M+K]^+$	I	Sugars and glycosides
7	1.354	4–aminobutanoate	$C_4H_9NO_2$	–0.47	104.0706	$[M+H]^+$	II	Nitrogen-containing compounds
8	1.357	trigonelline	$C_7H_7NO_2$	–0.28	138.0549	$[M+H]^+$	I	Nitrogen-containing compounds
9	1.360	guanine	$C_5H_5N_5O$	–0.20	152.0567	$[M+H]^+$	II	Nitrogen-containing compounds
10	1.365	pyroglutamic acid	$C_5H_7NO_3$	0.13	130.0499	$[M+H]^+$	II	Nitrogen-containing compounds
11	1.391	succinic acid	$C_4H_6O_4$	–1.03	117.0192	$[M–H]^–$	I	Organic acids
12	1.411	4–(hexopyranosyloxy)–3–methoxybenzoic acid	$C_{14}H_{18}O_9$	–0.48	329.0877	$[M–H]^–$	II	Phenolic acids
13	1.426	2,3–dihydroxy–2–methylbutanoic acid	$C_5H_{10}O_4$	–1.18	179.0560	$[M+FA–H]^–$	II	Organic acids
14	1.531	gallic acid	$C_7H_6O_5$	–0.78	169.0141	$[M–H]^–$	I	Phenolic acids
15	1.608	4–hydroxy–3–methoxyphenyl β–d–glucopyranoside	$C_{13}H_{18}O_8$	–0.69	301.0927	$[M–H]^–$	II	Sugars and glycosides
16	1.615	adenosine	$C_{10}H_{13}N_5O_4$	–0.44	268.1039	$[M+H]^+$	II	Nitrogen-containing compounds
17	1.620	adenine	$C_5H_5N_5$	–0.14	136.0618	$[M+H]^+$	I	Nitrogen-containing compounds
18	1.623	guanosine	$C_{10}H_{13}N_5$	–0.46	284.0988	$[M+H]^+$	I	Nitrogen-

			O_5					containing compounds
19	1.770	L–leucine	$C_6H_{13}NO_2$	–1.10	132.1018	$[M+H]^+$	II	Nitrogen-containing compounds
20	1.807	2–pyrrolidinecarboxylic acid	$C_5H_9NO_2$	–0.35	116.0706	$[M+H]^+$	II	Nitrogen-containing compounds
21	1.959	5–(hydroxymethyl)–2–furaldehyde	$C_6H_6O_3$	–0.06	127.0390	$[M+H]^+$	II	Other constituents
22	2.171	gentisic acid 5–O–β–glucoside	$C_{13}H_{16}O_9$	–0.62	315.0720	$[M–H]^–$	II	Phenolic acids
23	2.181	cinnamic acid	$C_9H_8O_2$	–0.88	166.0861	$[M+NH4]^+$	II	Phenylpropanoid derivatives
24	2.703	3,4–dimethoxyphenol	$C_8H_{10}O_3$	–1.34	153.0555	$[M–H]^–$	II	Phenolic acids
25	2.790	protocatechuic acid	$C_7H_6O_4$	–1.28	153.0191	$[M–H]^–$	I	Phenolic acids
26	2.815	syringic acid	$C_9H_{10}O_5$	–0.58	197.0454	$[M–H]^–$	I	Phenolic acids
27	2.827	glucosyringic acid	$C_{15}H_{20}O_{10}$	–1.40	359.0978	$[M–H]^–$	I	Phenolic acids
28	3.030	chlorogenic acid	$C_{16}H_{18}O_9$	–1.83	353.0872	$[M–H]^–$	II	Phenolic acids
29	3.171	caftaric acid	$C_{13}H_{12}O_9$	–1.15	311.0405	$[M–H]^–$	I	Phenolic acids
30	3.286	M–coumaric acid	$C_9H_8O_3$	–0.43	165.0546	$[M+H]^+$	II	Phenylpropanoid derivatives
31	3.407	esculin	$C_{15}H_{16}O_9$	–1.44	339.0716	$[M–H]^–$	II	Phenylpropanoid derivatives
32	3.449	procyanidin b1	$C_{30}H_{26}O_{12}$	–1.11	577.1344	$[M–H]^–$	II	Flavonoids
33	3.460	gallocatechin	$C_{15}H_{14}O_7$	–0.98	305.0664	$[M–H]^–$	I	Flavonoids
34	3.532	L–isoleucyl–L–proline	$C_{11}H_{20}N_2O_3$	–0.43	229.1546	$[M+H]^+$	II	Nitrogen-containing compounds
35	3.533	indoleacrylic acid	$C_{11}H_9NO_2$	–0.68	188.0705	$[M+H]^+$	II	Nitrogen-containing compounds
36	3.685	taxifolin 7–O–glucoside	$C_{21}H_{22}O_{12}$	–0.98	465.1033	$[M–H]^–$	I	Flavonoids
37	3.686	taxifolin	$C_{15}H_{12}O_7$	–0.41	305.0656	$[M+H]^+$	I	Flavonoids
38	3.713	3–caffeoylquinic acid	$C_{16}H_{18}O_9$	–0.09	355.1023	$[M+H]^+$	I	Phenylpropanoid derivatives
39	3.843	quercetin 3,7–diglucoside	$C_{27}H_{30}O_{17}$	–0.28	625.1406	$[M–H]^–$	I	Flavonoids
40	3.943	quinic acid	$C_7H_{12}O_6$	–0.90	191.0559	$[M–H]^–$	I	Organic acids
41	3.973	(+)–hypaphorine	$C_{14}H_{18}N_2O_2$	–0.13	247.1441	$[M+H]^+$	II	Nitrogen-containing compounds
42	4.082	3–hydroxycinnamic	$C_9H_8O_3$	–1.08	163.0399	$[M–H]^–$	I	Phenylpropanoid

		acid						derivatives
43	4.217	procyanidin b2	$C_{30}H_{26}O_{12}$	–1.42	577.1343	$[M–H]^{–}$	II	Flavonoids
44	4.456	4–hydroxy–3–methoxycinnamic acid	$C_{10}H_{10}O_4$	–0.66	193.0505	$[M–H]^{–}$	II	Phenylpropanoid derivatives
45	4.536	2–(3,4–dihydroxyphenyl)–7–hydroxy–3,4–dihydro–2H–1–benzopyran–4–one	$C_{15}H_{12}O_5$	–0.13	273.0757	$[M+H]^{+}$	II	Flavonoids
46	4.541	4–hydroxybenzoic acid	$C_7H_6O_3$	–0.55	139.0389	$[M+H]^{+}$	II	Phenylpropanoid derivatives
47	4.541	benzoic acid	$C_7H_6O_2$	–0.66	123.0440	$[M+H]^{+}$	I	Organic acids
48	4.555	cyanidanol	$C_{15}H_{14}O_6$	–1.22	289.0714	$[M–H]^{–}$	II	Flavonoids
49	4.683	6–hydroxykaempferol 3–rutinoside–6–glucoside	$C_{33}H_{40}O_{21}$	–0.03	771.1989	$[M–H]^{–}$	I	Flavonoids
50	4.745	procyanidin c1	$C_{45}H_{38}O1_{8}$	–0.94	865.1979	$[M–H]^{–}$	I	Flavonoids
51	4.747	2–hydroxynaringenin 4′–O–glucopyranoside	$C_{21}H_{22}O_{11}$	–1.01	449.1085	$[M–H]^{–}$	I	Flavonoids
52	4.769	3–p–coumaroylquinic acid	$C_{16}H_{18}O_8$	–0.53	337.0927	$[M–H]^{–}$	I	Phenylpropanoid derivatives
53	4.846	5–(4–hydroxypentyl)–1,3–benzenediol	$C_{11}H_{16}O_3$	–0.25	197.1172	$[M+H]^{+}$	I	phenols
54	4.847	cryptochlorogenic acid	$C_{16}H_{18}O_9$	–1.83	353.0872	$[M–H]^{–}$	II	Phenylpropanoid derivatives
55	4.894	sucrose	$C_{12}H_{22}O_{11}$	–1.26	341.1085	$[M–H]^{–}$	II	Sugars and glycosides
56	4.985	myricetin 3–rutinoside	$C_{27}H_{30}O_{17}$	–0.42	625.1407	$[M–H]^{–}$	I	Flavonoids
57	5.067	hibifolin	$C_{21}H_{18}O_{14}$	–0.63	493.0620	$[M–H]^{–}$	I	Flavonoids
58	5.089	3–O–caffeoylquinic acid methyl ester	$C_{17}H_{20}O_9$	–1.25	367.1030	$[M–H]^{–}$	I	Phenylpropanoid derivatives
59	5.115	myricetin	$C_{15}H_{10}O_8$	–0.14	319.0448	$[M+H]^{+}$	I	Flavonoids
60	5.137	myricetin 3–O–β–d–galactopyranoside	$C_{21}H_{20}O_{13}$	–0.67	479.0827	$[M–H]^{–}$	I	Flavonoids
61	5.317	1–caffeoylquinic acid	$C_{16}H_{18}O_9$	–0.69	353.0876	$[M–H]^{–}$	I	Phenylpropanoid derivatives
62	5.529	rutin	$C_{27}H_{30}O_{16}$	–1.33	609.1454	$[M–H]^{–}$	I	Flavonoids
63	5.531	scutellarein	$C_{15}H_{10}O_6$	–0.08	287.0550	$[M+H]^{+}$	I	Flavonoids
64	5.556	L–phenylalanine	$C_9H_{11}NO_2$	–1.02	164.0715	$[M–H]^{–}$	I	Nitrogen-containing compounds
65	5.689	syringaldehyde	$C_9H_{10}O_4$	–0.59	183.0651	$[M+H]^{+}$	I	phenols
66	5.748	quercetin	$C_{15}H_{10}O_7$	–0.53	301.0352	$[M–H]^{–}$	II	Flavonoids
67	6.034	guaijaverin	$C_{20}H_{18}O_{11}$	–0.77	433.0773	$[M–H]^{–}$	I	Flavonoids

68	6.298	kaempferol 3–O–β–d–glucuronide	$C_{21}H_{18}O_{12}$	–0.28	461.0724	$[M–H]^{–}$	I	Flavonoids
69	6.348	syringetin 3–O–galactoside	$C_{23}H_{24}O_{13}$	0.05	507.1144	$[M–H]^{–}$	I	Flavonoids
70	6.350	trifolin	$C_{21}H_{20}O_{11}$	–0.19	447.0932	$[M–H]^{–}$	I	Flavonoids
71	6.389	isorhamnetin 3–O–glucoside	$C_{22}H_{22}O_{12}$	–1.02	477.1034	$[M–H]^{–}$	I	Flavonoids
72	6.488	miquelianin	$C_{21}H_{18}O_{13}$	–1.50	479.0813	$[M+H]^{+}$	I	Flavonoids
73	6.556	isoquercitrin	$C_{21}H_{20}O_{12}$	–0.40	463.0880	$[M–H]^{–}$	I	Flavonoids
74	6.604	butein	$C_{15}H_{12}O_{5}$	–0.15	271.0612	[M–H]–1	I	Flavonoids
75	6.662	morin	$C_{15}H_{10}O_{7}$	–0.92	303.0497	$[M+H]^{+}$	I	Flavonoids
76	6.738	eriodictyol 7–O–glucoside	$C_{21}H_{22}O_{11}$	–0.11	449.1089	$[M–H]^{–}$	I	Flavonoids
77	6.881	kaempferol 3–O–sophoroside	$C_{26}H_{28}O_{15}$	–0.33	581.1499	$[M+H]^{+}$	I	Flavonoids
78	7.078	kaempferol 3–O–rutinoside	$C_{27}H_{30}O_{15}$	–0.27	595.1656	$[M+H]^{+}$	I	Flavonoids
79	7.331	quercetin 3–O–[6′′–O–(3–hydroxy–3–methylglutaroyl)–β–d–galactoside]	$C_{27}H_{28}O_{16}$	–0.19	609.1449	$[M+H]^{+}$	I	Flavonoids
80	7.369	isorhamnetin 3–O–neohesperidoside	$C_{28}H_{32}O_{16}$	–0.26	625.1762	$[M+H]^{+}$	I	Flavonoids
81	7.944	loliolide	$C_{11}H_{16}O_{3}$	–0.17	197.1172	$[M+H]^{+}$	II	Other constituents
82	7.994	spinatoside	$C_{23}H_{22}O_{14}$	–0.38	523.1080	$[M+H]^{+}$	II	Flavonoids
83	8.005	isorhamnetin 3–glucuronide	$C_{22}H_{20}O_{13}$	–0.46	493.0974	$[M+H]^{+}$	II	Flavonoids
84	8.120	coniferyl aldehyde	$C_{10}H_{10}O_{3}$	–0.54	179.0702	$[M+H]^{+}$	II	Phenylpropanoid derivatives
85	8.123	eriodictyol	$C_{15}H_{12}O_{6}$	–0.06	289.0707	$[M+H]^{+}$	I	Flavonoids
86	8.125	quercitrin	$C_{21}H_{20}O_{11}$	–0.04	449.1078	$[M+H]^{+}$	I	Flavonoids
87	11.550	DL–proline	$C_{5}H_{9}NO_{2}$	–0.55	116.0705	$[M+H]^{+}$	II	Nitrogen-containing compounds
88	14.978	procyanidin	$C_{30}H_{26}O_{13}$	–1.38	595.1438	$[M+H]^{+}$	I	Flavonoids
89	16.085	phytosphingosine	$C_{18}H_{39}NO_{3}$	–0.12	318.3002	$[M+H]^{+}$	II	Nitrogen-containing compounds
90	16.841	α–linolenic acid	$C_{18}H_{30}O_{2}$	–0.27	279.2318	$[M+H]^{+}$	I	Other constituents
91	18.01	erucamide	$C_{22}H_{43}NO$	–0.11	338.3417	$[M+H]^{+}$	II	Nitrogen-

	1							containing compounds
92	18.13 3	linoleoyl ethanolamide	$C_{20}H_{37}NO_2$	–0.20	324.2896	$[M+H]^+$	II	Nitrogen-containing compounds
93	18.14 7	palmitamide	$C_{16}H_{33}NO$	–1.09	256.2632	$[M+H]^+$	II	Nitrogen-containing compounds
94	18.22 9	1–linolenoylglycerol	$C_{21}H_{36}O_4$	–0.23	353.2686	$[M+H]^+$	II	Other constituents
95	18.81 3	sphingosine	$C_{18}H_{37}NO_2$	–0.11	300.2897	$[M+H]^+$	II	Nitrogen-containing compounds
96	18.84 4	1–monolinolein	$C_{21}H_{38}O_4$	–1.16	355.2839	$[M+H]^+$	II	Other constituents

I: Confidence Level I: The I series of compounds were clearly identified by matching theMS/MS spectra with the commercially available true reference standards analyzed under the same conditions.

II: Confidence level II: The annotation of the compound was only based on the database-derived MS/MS fragment matching results, and the reference standard was not used.

Table S2. Twelve central targets obtained from the pathway-overlap analysis.

**Gene**	**UniProt ID**	**Protein**	**Network context**
ADORA2A	P29274	Adenosine receptor A2A	Receptor/cAMP signalling
CAMK2B	Q13554	Calcium/calmodulin-dependent protein kinase II β	Calcium-dependent kinase
DRD2	P14416	Dopamine D2 receptor	Gi-coupled receptor
EDNRA	P25101	Endothelin receptor type A	G-protein-coupled receptor
ADCY5	O95622	Adenylate cyclase 5	cAMP generation
PRKACA	P17612	cAMP-dependent protein kinase catalytic subunit α	cAMP effector
PTGER2	P43116	Prostaglandin E2 receptor EP2	Gs-coupled receptor
F2R	P25116	Proteinase-activated receptor 1	G-protein-coupled receptor
PTGER3	P43115	Prostaglandin E2 receptor EP3	Gi/Gs-coupled receptor
CHRM2	P08172	Muscarinic acetylcholine receptor M2	Gi-coupled receptor
CHRM1	P11229	Muscarinic acetylcholine receptor M1	Gq-coupled receptor
GRIN1	Q05586	Glutamate receptor ionotropic, NMDA 1	Ionotropic receptor

Table S3. Network centrality of the eight prioritised compounds.

Candidate compound	BC	CC	DC
5-(4-hydroxypentyl)-1,3-benzenediol	21.45	0.47	3.00
(+)-hypaphorine	16.80	0.45	3.00
adenosine	15.97	0.46	3.00
α-linolenic acid	13.75	0.45	3.00
quinic acid	9.16	0.46	3.00
taxifolin	9.16	0.46	3.00
quercetin	9.16	0.46	3.00
butein	9.16	0.46	3.00

BC, betweenness centrality; CC, closeness centrality; DC, degree centrality.

Table S4. RMSD values and radius of gyration (Rg) of the ligands in six simulated systems.

System	Ligand RMSD relative to binding site (mean/max, Å)	Ligand internal RMSD (mean, Å)	Rg (mean, Å)
acarbose–α-amylase	10.62 / 15.80	3.21	5.50
butein–α-amylase	3.32 / 6.98	0.89	4.35
acarbose–α-glucosidase	14.59 / 31.03	3.02	5.46
butein–α-glucosidase	3.07 / 6.58	0.65	4.28
acarbose–DRD2	5.15 / 8.01	2.18	5.89
butein–DRD2	5.46 / 8.31	0.95	4.33

Mean and maximum values are given for the ligand RMSD relative to the binding site (fit to the protein backbone); the ligand internal RMSD is computed relative to its initial bound conformation. Rg denotes the mean radius of gyration over the trajectory.

Table S5. Representative protein–ligand contacts and their occupancies (%) over the trajectory in six simulated systems.

System	Stable H-bonds (≥30%)	π–π stacking	π–cation	Main water bridges
acarbose–α-amylase	Asp402 (91%), Thr6 (78%), Gly403 (62%)	—	—	Asp402 (59%)
butein–α-amylase	Asp300 (79%), Asp356 (67%)	Trp59 (78%), Trp58 (23%)	—	Gln63 (33%)
acarbose–α-glucosidase	None (max ≈ 21%)	—	—	Glu792 (33%)
butein–α-glucosidase	Ile759 (96%), Glu792 (59%), Arg676 (44%)	Tyr659 (37%)	Arg699 (29%)	Glu301 (40%)
acarbose–DRD2	Asp114 (89%), Asn396 (45%), Ile184 (44%)	—	—	Cys182 (47%)
butein–DRD2	Gln63 (35%)	Trp59 (42%)	—	Gln63 (18%)

Hydrogen bonds with an occupancy ≥ 30% are reported as stable; π–π stacking and π–cation (cation–π) interactions involved in the binding interface are listed where present, together with the major water-bridge contacts. "None" indicates that no residue reached the 30% threshold.
